# Synthesis and In Vitro Pharmacological Evaluation of 5,8-Dideaza Analogs of Methotrexate

**DOI:** 10.3390/molecules30132772

**Published:** 2025-06-27

**Authors:** Marta Abellán-Flos, Charles Skarbek, Dáire J. Gibbons, Estelle Rascol, Ainhoa García, Raphaël Labruère

**Affiliations:** 1 Institut de Chimie Moléculaire et des Matériaux d’Orsay, Université Paris-Saclay, CNRS UMR 8182, 91400 Orsay, Franceestelle.rascol@u-bordeaux.fr (E.R.); ainhoa.garcia-martin@curie.fr (A.G.); 2 Chimie et Biologie des Membranes et des Nanoobjets, Bordeaux INP, Université de Bordeaux, CNRS UMR 5248, 33600 Pessac, France; 3 Institut Curie, Université Paris-Saclay, CNRS UMR9187, Inserm U1196, 91400 Orsay, France; 4 Institut Curie, Université PSL, CNRS UMR9187, Inserm U1196, 91400 Orsay, France

**Keywords:** methotrexate, quinazoline, cytotoxicity, DHFR, in vitro metabolism

## Abstract

This study describes the synthesis of a series of dideaza analogs of methotrexate and their preliminary pharmacological and metabolic evaluation. The 5,8-dideazamethotrexate was efficiently obtained in five steps using a new synthetic route. Oxygenated and thiolated analogs of dideazamethotrexate were prepared following the devised pathway. Their cytotoxicity was studied in the A549 lung cancer cell line, as well as their DHFR dihydrofolate reductase inhibition activity and in vitro metabolism. The two new analogs showed strong activity on cancer cells and the enzymatic target. These compounds were not metabolized, a clear advantage over methotrexate, which is rapidly converted to the toxic metabolite 7-hydroxymethotrexate.

## 1. Introduction

**Figure 1:**
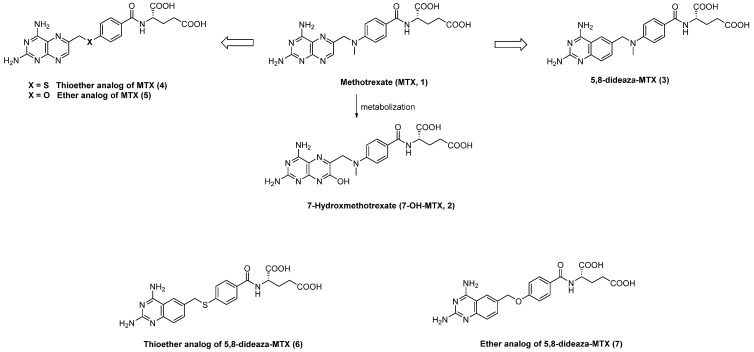
Methotrexate, 7-hydroxymethotrexate, and analogs of methotrexate.

Methotrexate (MTX, **1**, Figure 1) is an antimetabolite and antifolate drug used in human and veterinary medicine to treat rheumatism and cancer [1,2,3]. Methotrexate acts as a potent inhibitor of dihydrofolate reductase (DHFR), which is responsible for purine and pyrimidine synthesis in cells [4,5]. Although widely prescribed, this molecule has certain drawbacks, such as the development of resistances and the production of a toxic metabolite in humans. Research focused on the discovery of novel classical antifolate drugs have been actively pursued, which has led to the identification of numerous derivatives showing promising therapeutic properties [6,7,8,9].

Regarding its human metabolism, methotrexate is subject to liver transformation that can generate 7-hydroxymethotrexate (7-OH-MTX, **2**, Figure 1) via aldehyde oxidase action [10,11]. This metabolite is toxic towards the liver and the kidney, and does not improve the drug’s efficacy [12,13]. Kidney toxicity is due to the low water solubility of 7-OH-MTX (**2**), leading to its precipitation in the nephrons [14].

As part of a program to develop new MTX analogs, we were seeking to identify effective molecules that do not metabolize rapidly. Previous findings have demonstrated that the MTX analog bearing a 2,4-diaminoquinazoline instead of a 2,4-diaminopteridine moiety (such as **3**, Figure 1) inhibits DHFR with the same binding affinity and presents slightly increased cytotoxic activity against cancer cells [15,16]. Moreover, two analogs of methotrexate containing either an ether or a thioether bond between the pteridine nucleus, and the benzoyl-glutamyl part instead of the *N*-methyl group (**4** and **5**, Figure 1), were shown to display strong anticancer activity in vitro and in vivo [17,18,19]. Regarding these analogs, the hydroxylation of position 7 of the pteridine nucleus was also observed in varying proportions using in vitro metabolism assays [20].

As the quinazoline ring differs significantly from the pteridine ring, the troublesome hydroxylation by the aldehyde oxidase seen in methotrexate, or analogs bearing the pteridine ring, should not happen. We, therefore, decided to prepare analogs **6** and **7** that would benefit from the two structural modifications: a dideaza ring and a heteroatom replacing the *N*-methyl moiety.

The oxygenated and thiolated analogs of dideazamethotrexate were then prepared and their cytotoxicity was assessed on the A549 lung cancer cell line. Inhibition activity on DHFR and their in vitro metabolism was further evaluated. Their metabolization was studied directly on aldehyde oxidase from rabbit liver, on cytosol from human liver, and on the liver S9 fraction.

## 2. Results and Discussion

### 2.1. Synthesis

**Scheme 1:**
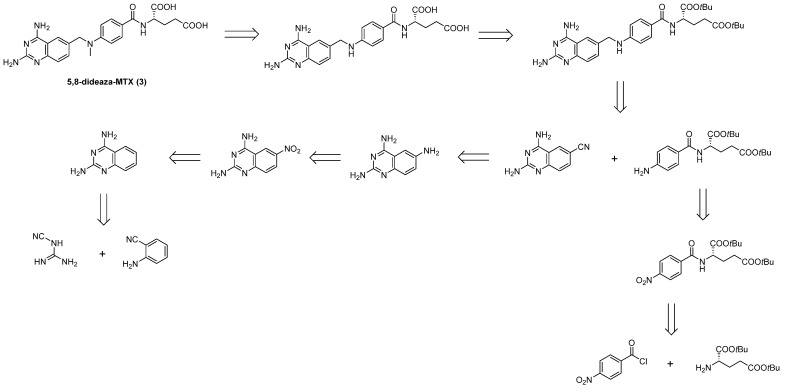
The described route for the preparation of 5,8-dideaza MTX analog (**3**): convergent synthesis from quinazoline and aminobenzoylglutamate precursors obtained in four and two steps, respectively [15,21].

The synthesis of the 5,8-dideaza analog of MTX (**3**) was previously described [15,21] (Scheme 1): Briefly, analog **3** was globally generated from the coupling of a quinazoline motif and an aminobenzoylglutamate. From these reports, the reactive quinazoline was prepared in four steps while the aniline partner was obtained in two steps.

Herein, we decided to devise a new synthetic pathway that would be adapted for the syntheses of all the designed analogs. Instead of connecting the quinazoline moiety to the benzoyl glutamyl part, our approach involved initially coupling a quinazoline precursor to various benzoyl glutamyl scaffolds and subsequently forming the quinazoline core (Scheme 1).

First, we synthesized the 5-(bromomethyl)-2-fluorobenzonitrile **9**, the common intermediate for the preparation of the analogs. Starting from 2-fluoro-5-methylbenzonitrile **8**, a Wohl–Ziegler reaction was carried out using a described procedure with azobisisobutyronitrile (AIBN) as a radical initiator and *N*-bromosuccinimide (NBS) as the source of bromine radicals [22,23] and afforded **9** in a 52% yield. A nucleophilic substitution between bromobenzyl **9** and the previously prepared *N*-methyl aniline **10** was achieved in dry DMF at room temperature and in the presence of anhydrous K_2_CO_3_ to give **11** in an almost quantitative yield. The 2,4-diaminoquinazoline **12** was formed by reacting the benzonitrile **11** with guanidine carbonate in DMF at 150 °C [24]. The 5,8-dideaza analog of MTX (**3**) was then obtained by the deprotection of the *tert*-butyl groups of **12** with TFA in 85% yield. Analog **3** was, therefore, prepared as the TFA salt in five steps in a ~31% yield, which includes the preparation of the intermediate **10** in one step. This synthesis was more efficient and concise than the one previously reported in nine steps with an overall yield of 12% [15,21].

**Scheme 2:**
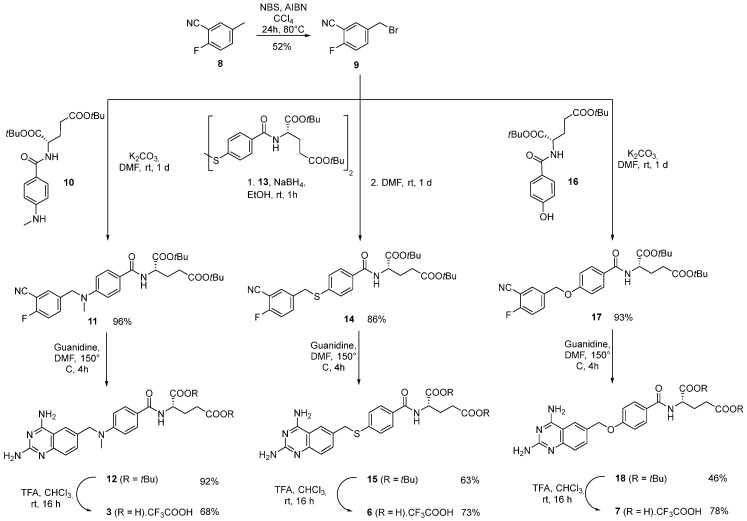
Synthetic pathway to 5,8-dideaza analogs of MTX.

Analogs **6** and **7**, containing either a thioether or an ether bond, were prepared following the same synthetic pathways (Scheme 2). Each new analog is derived from suitable phenyl-glutamate precursors **13** and **16** that were previously synthesized [20]. At this stage, similar procedures were applied to the two other analogs, except that the disulfide bond of intermediate **13** was first reduced to yield the free thiol, which then substituted the bromine in benzonitrile **9**. The thioanalog **6** (TFA salt) was further obtained in an overall yield of ~20%. Regarding the synthesis of ether analog **7**, phenol **16** was used as the building block, and this route afforded compound **7** (TFA salt) in an overall yield of ~17%.

### 2.2. In Vitro Cytotoxicity and Enzymatic Inhibition Activity

The in vitro cytotoxicity (IC_50_) of the three analogs was evaluated on human lung carcinoma (A549) cells. This cell line has been widely used to ascertain the cytotoxicity of MTX in numerous studies [20,25,26,27]. This provides a strong basis for comparison with previously published data obtained with this cell line on MTX and analogs. The compounds were incubated in the presence of cells for 72 h and the results are depicted in Table 1. Methotrexate was used as a reference in this study, and we observed an IC_50_ of 0.12 µM, a slightly higher value than previously obtained [25]. The dideaza analog of MTX (**3**) was slightly more active (by a factor of three) than the parent compound **1**, in line with what was observed earlier [15]. The thioether derivative of dideazamethotrexate **6** resulted in no improvement of the antiproliferative activity as compared to dideazamethotrexate **3** but the introduction of the oxygen atom produced the most cytotoxic derivative **7** of this series with an IC_50_ value of 0.02 µM. Notably, quinazolines **6** and **7** were approximately ten times more cytotoxic than their pteridine counterparts **4** and **5**, with IC₅₀ values of 0.31 µM and 2.60 µM, respectively [20].

To further understand the mechanism of action of the new analogs, we tested their ability to inhibit the enzyme dihydrofolate reductase (DHFR), the pharmacological target of MTX (**1**). As a competitive inhibitor of DHFR, MTX (**1**) impedes its ability to catalyze the reversible NADPH-dependent reduction of dihydrofolic acid to tetrahydrofolic acid, a coenzyme involved in nucleic acid synthesis.

The IC_50_ values of the three analogs and MTX (**1**) were determined using a spectrophotometric assay (Table 1). MTX (**1**) has an IC_50_ of 30 nM, which is consistent with values found in the literature [15,28]. Analog **3** achieved the highest enzyme inhibition with an IC_50_ of 7 nM. The other two analogs, **6** and **7**, had an IC_50_ intermediate between those of MTX (**1**) and **3**. Overall, all the molecules are very good DHFR inhibitors, with inhibition capacities close to each other.

The ether analog **7** with the highest cytotoxic activity is also the one with the lowest DHFR inhibition of the quinazoline series. This may be explained by the fact that MTX and its derivatives exert cytotoxic activity by targeting other enzymes, such as thymidylate synthase (TYMS) [29].

### 2.3. In Vitro Metabolism

Next, we conducted in vitro hepatic metabolism assays on methotrexate and its analogs and further identification of parent compounds and metabolites was carried out using HPLC-MS analysis. Methotrexate undergoes liver metabolism, resulting in the formation of 7-hydroxymethotrexate due to the action of aldehyde oxidase [10,11]. Partially purified aldehyde oxidase extracted from rabbit liver was used to determine the potential hydroxylation of the different analogs. After 24 h of incubation, we first verified that MTX (**1**) was converted into the expected 7-hydroxymethotrexate (**2**). The analogs were incubated in the same conditions and no hydroxylation or any other transformation of the parent compounds were observed. This result confirmed that the quinazoline ring prevents hydroxylation at position 7, either because of the different reactivity of the ring, or because these analogs are not substrates for this enzyme.

In order to examine other possible metabolization of the analogs, we tested their stability in a more complex system: the cytosol from human liver. Human liver cytosol contains various soluble enzymes that metabolize drugs and is usually employed for predicting metabolic activity. Methotrexate was used as a positive control since aldehyde oxidase is also present in the cytosol from the liver and the 7-hydroxymethotrexate (**2**) was again observed as the sole metabolite in this experiment. We studied the dideaza analogs **3**, **6**, and **7** in the same assay and no transformation was detected after 28 h of incubation.

Eventually, the different compounds were incubated with human pooled S9 fractions. Liver S9 contains both microsomal and cytosolic fractions of liver cells and, therefore, this experiment provides essential information on the transformation of phase I and II metabolism. Apart from methotrexate, which was metabolized into **2**, the other compounds remained intact even in the presence of the NADPH cofactor during incubation at 37 °C for up to 12 h (see Supplementary Materials). In particular, the oxidation-susceptible thioether motif of analog **6** was not metabolized, which could be due to the decreased reactivity of the sulfur atom caused by the electron-withdrawing effect of the benzoyl group through resonance [30].

In summary, these derivatives showed no in vitro metabolization using aldehyde oxidase, cytosol from human liver, and human pooled S9 fractions. In particular, no hydroxylation was observed on the quinazoline ring as opposed to the pteridine ring of methotrexate.

## 3. Materials and Methods

### 3.1. Synthesis

All chemical reagents were obtained from Acros, Aldrich, Alfa Aesar, or Fluorochem, and were used without further purification. Solvents were obtained from SDS, VWR-Prolabo, or Carlo Erba. Flash chromatography was performed using silica gel (35–70 μm, Merck, Darmstadt, Germany). Evaporation of solutions was performed under reduced pressure at temperature below 40 °C using a rotary evaporator. Analytical TLC was performed using Silica Gel 60 F254 pre-coated aluminum plates (Merck). Spots were visualized by absorbance/emission under UV light at 254 or 360 nm. NMR spectra were collected on Bruker DPX 250 (^1^H at 250 MHz and ^13^C at 62.5 MHz), AV 300 (^1^H at 300 MHz and ^13^C at 75 MHz), or AV 360 (^1^H at 360 MHz and ^13^C at 90 MHz) spectrometers and analyzed using MestReNova software version 12.0.0. Chemical shifts are reported in ppm (δ) and coupling constants in Hz (J). NMR spectra were performed in CDCl_3_ or (CD_3_)_2_SO. High-resolution mass spectrometry (HRMS) analyses were performed on a Bruker MicroTOF-Q apparatus by electrospray with positive or negative (ESI^+^ or ESI^−^). The purity of all compounds used for the biological activity test was checked by reverse phase analytical HPLC and confirmed to be ≥95%. Preparation of intermediates **10**, **13,** and **16** was previously reported [20].

#### 3.1.1. Compound **9**: 5-(Bromomethyl)-2-fluorobenzonitrile

2-Fluoro-5-methylbenzonitrile **8** (1.00 g, 1.00 equiv., 7.40 mmol) was dissolved in carbon tetrachloride (35 mL) at room temperature under argon atmosphere. To this colorless solution, *N*-bromosuccinimide (1.38 g, 1.05 equiv., 7.77 mmol) and then 2,2′-azobis(2-methylpropionitrile) (122 mg, 0.10 equiv., 0.74 mmol) were added. The resulting heterogeneous mixture was refluxed at 80 °C for 23 h and was then allowed to cool to room temperature and filtered. The filtrate was concentrated by evaporation under reduced pressure to give a pale-yellow crude residue that was purified by flash chromatography (cyclohexane:EtOAc, 85:15) to afford 5-(bromomethyl)-2-fluorobenzonitrile **9** (827 mg, 3.86 mmol, 52%) as a white solid. The analytical data of **9** were in complete agreement with the literature data [28].

R*f *0.5 (cyclohexane:EtOAc, 85:15); mp 78 °C; ^1^H NMR (300 MHz, CDCl_3_) δ ppm: 7.73–7.58 (m, 2H, *CH*_Ar_), 7.20 (t, *J* = 8.5 Hz, 1H, *CH*_Ar_-CF), 4.44 (s, 2H, CH_2_); ^13^C NMR (75MHz, CDCl_3_) δ ppm: 161.1 (*C*F), 135.9 (*CH*_Ar_, d, *J *= 8.25 Hz), 135.3 (*CH*_Ar_CH_2_), 134.0 (*CH*_Ar_), 117.2 (*CH*_Ar_, d, *J *= 20.25 Hz), 113.5 (*CH*_Ar_CN), 102.1 (*C*N, d, *J *= 15 Hz), 30.5 (*CH*_2_); MS ESI^+^: [M+H]^+^ 214.0.1, [M+Na]^+^ 236.0. HRMS: (ESI^+^-MS, *m*/*z*) calcd for C_8_H_6_BrFN [M+H]^+^: 213.9624, found: 213.9630.

#### 3.1.2. Compound **11**: Di-*tert*-butyl (4-((3-cyano-4-fluorobenzyl)(methyl)amino)benzoyl)-*L*-glutamate

Compound **10** [20] (724 mg, 1.00 equiv., 1.845 mmol) and **9** (395 mg, 1.00 equiv., 1.845 mmol) were dissolved in anhydrous DMF (4.5 mL). Potassium carbonate (510 mg, 2.00 equiv., 3.69 mmol) was added and the mixture was stirred at room temperature under Ar for 1 day. Then, the mixture was diluted with EtOAc, followed by washing with water and brine. The organic phase was dried over MgSO_4_, filtered, and evaporated under reduced pressure. The crude product was purified by flash chromatography (cyclohexane/EtOAc, 8/2) to provide **11** (905 mg, 1.72 mmol, 93%) as a white sticky solid.

R*f *0.64 (cyclohexane:EtOAc, 6:4); mp 60 °C; ^1^H NMR (300 MHz, CDCl_3_) δ ppm: 7.73–7.67 (m, 2H, 2× C*H*_Ar_), 7.43–7.39 (m, 2H, 2× C*H*_Ar_), 7.16 (t, *J* = 8.9 Hz, 1H, CFC*H*_Ar_), 6.78 (d, *J* = 7.5 Hz, 1H, NH), 6.65 (d, *J* = 9.0 Hz, 2H, 2× C*H*_Ar_), 4.65 (td, *J* = 7.8, 4.6 Hz, 1H, NHC*H*), 4.57 (s, 2H, C_Ar_C*H*_2_), 3.09 (s, 3H, C*H*_3_), 2.45–2.14 (m, 3H, C*H*HCH_2_CO, CH_2_CO), 2.06–1.94 (m, 1H, C*H*HCH_2_CO), 1.47 (s, 9H, *C*(CH_3_)_3_), 1.39 (s, 9H, *C*(CH_3_)_3_). ^13^C NMR (75 MHz, CDCl_3_) δ ppm: 172.7 (*C*OO*t*Bu), 171.7 (*C*OO*t*Bu), 166.8 (*C*ONH), 164.1 (CF), 160.7 (CF), 151.3 (*C*_Ar_), 135.4 (*C*_Ar_), 133.3 (*CH*_Ar_, d, *J *= 8.25 Hz), 131.4 (*CH*_Ar_), 129.0 (2x*CH*_Ar_), 122.3 (CO*C*_Ar_), 117.0 (*CH*_Ar_, d, *J *= 19.5 Hz), 113.9 (*CH_Ar_*CN), 111.5 (2× *CH*_Ar_), 101.9 (CH_Ar_*CN*, d, *J *= 16.5 Hz), 82.4 (*C*(CH_3_)_3_), 80.8 (*C*(CH_3_)_3_), 55.2 (*CH*_2_C_Ar_), 52.7 (NH*CH*), 39.0 (CH_3_), 31.8 (CH_2_CO), 28.1 (C(*CH*_3_)_3_), 27.9 (CH_2_). MS ESI^+^: [M+H]^+^ 526.3, [M+Na]^+^ 548.3. HRMS: (ESI^+^-MS, *m*/*z*) calcd for C_29_H_36_FN_3_NaO_5_ [M+Na]^+^: 548.2531, found: 548.2521.

#### 3.1.3. Compound **12**: Di-*tert*-butyl (4-(((2,4-diaminoquinazolin-6-yl)methyl)(methyl)amino)benzoyl)-*L*-glutamate

Compound **11** (400 mg, 1.00 equiv., 0.78 mmol,) and guanidine carbonate (281 mg, 4.00 equiv., 3.12 mmol) were dissolved in anhydrous DMF (15 mL) and the mixture was heated at 150 °C for 4 h under Ar. The mixture was allowed to cool to room temperature and the solvent was evaporated under reduced pressure. The resulting residue was triturated with CH_2_Cl_2_. To remove any excess guanidine, the white precipitate was resuspended in 10 mL of CH_2_Cl_2_:MeOH, 9:1, then filtered. The filtrate, which contained the desired product, was concentrated under reduced pressure to yield compound **12** as an off-white solid (403 mg, 0.72 mmol, 92% yield).

R*f *0.60 (CH_2_Cl_2_:MeOH, 85:15); mp 82 °C; ^1^H NMR (300 MHz, DMSO-d_6_) δ ppm: 8.44 (d, *J* = 7.6 Hz, 1H, CO*NH*), 7.86 (s, 1H, C*H*_Ar_), 7.72 (d, *J* = 8.6 Hz, 2H, 2× C*H*_Ar_), 7.33 (d, *J* = 8.8, 1H, C*H*_Ar_), 7.14 (d, *J* = 8.5 Hz, 1H, C*H*_Ar_), 6.77 (d, *J* = 8.7 Hz, 2H, 2× C*H*_Ar_), 6.65 (bs, 2H, NH_2_), 5.88 (bs, 2H, NH_2_), 4.62 (s, 2H, OC*H*_2_), 4.29 (dd, *J* = 9.1, 5.1 Hz, 1H, NHC*H*), 3.11 (s, 3H, C*H*_3_), 2.30 (t, *J* = 7.4 Hz, 2H, CH_2_CO), 2.06–1.82 (m, 2H, C*H*_2_), 1.40 (s, 9H, C(*CH*_3_)_3_), 1.38 (s, 9H, C(*CH*_3_)_3_). ^13^C NMR (75 MHz, DMSO-d_6_) δ ppm: 171.6 (*C*OO*t*Bu), 171.5 (*C*OO*t*Bu), 166.5 (*C*ONH), 162.3 (*C*_Ar_), 160.7 (*C*_Ar_), 158.7 (*C*_Ar_), 151.8 (*C*_Ar_), 151.3 (*C*_Ar_), 131.5 (*CH*_Ar_), 129.9 (*C*_Ar_), 128.9 (2x*CH*_Ar_), 124.6 (*CH*_Ar_), 121.8 (*CH*_Ar_), 120.6 (*C*_Ar_), 110.9 (2x*CH*_Ar_), 110.1 (*C*_Ar_), 80.4 (*C*(CH_3_)_3_), 79.8 (*C*(CH_3_)_3_), 55.2 (*CH*_2_N), 52.3 (NH*CH*), 35.8 (CH_3_), 31.4 (CH_2_CO), 27.8 (C(*CH*_3_)_3_), 26.1 (CH_2_). MS ESI^+^: [M+H]^+^ 565.3, [M+Na]^+^ 587.3. HRMS: (ESI^+^-MS, *m*/*z*) calcd for C_30_H_40_N_6_NaO_5_ [M+Na]^+^: 587.2952, found: 587.2929.

#### 3.1.4. Compound **3**: (4-(((2,4-Diaminoquinazolin-6-yl)methyl)(methyl)amino)benzoyl)-*L*-glutamic Acid TFA Salt

To a solution of **12** (400 mg, 1.00 equiv., 0.71 mmol) in CHCl_3_ (20 mL) under Ar, TFA (10 mL, 180 equiv., 127 mmol) was added dropwise and the reaction mixture was then stirred at room temperature for 16 h. The solvent was removed under reduced pressure, co-evaporated twice with MeOH (25 mL) and the brown residue was triturated with Et_2_O (5 mL) to provide a pale-yellow solid. The crude product was filtered with Et_2_O (30 mL) and 60 mL of CH_2_Cl_2_:MeOH, 8:2 to afford **3**.CF_3_COOH (272 mg, 0.48 mmol, 68%) as off-white solid.

mp > 260 °C; ^1^H NMR (400 MHz, DMSO-d_6_) δ ppm: 8.18 (s, 2H, N*H*_2_), 8.00 (s, 1H, C*H*_Ar_), 7.71 (d, *J* = 8.8 Hz, 2H, 2× C*H*_Ar_), 7.49 (d, *J* = 8.8 Hz, 1H, C*H*_Ar_), 7.34–7.28 (m, 3H, C*H*_Ar_ + N*H*_2_), 6.74 (d, *J* = 9.1 Hz, 2H, 2× C*H*_Ar_), 4.65 (s, 2H, NC*H*_2_), 4.31 (dd, *J* = 13.5, 7.0 Hz, 1H, NHC*H*), 3.12 (s, 3H, C*H*_3_), 2.29 (t, *J* = 8.0 Hz, 2H, C*H*_2_COO), 2.09–1.87 (m, 2H, CH_2_). ^13^C NMR (75 MHz, DMSO-d_6_) δ ppm: 174.3 (*C*OOH), 173.1 (*C*OOH), 166.6 (*C*OHN), 163.1 (*C*_Ar_), 155.0 (*C*_Ar_), 151.1 (*C*_Ar_), 138.7 (*C*_Ar_), 135.1 (*C*_Ar_), 134.3 (*CH*_Ar_), 129.0 (2× *CH*_Ar_), 122.8 (*CH*_Ar_), 121.1 (*C*_Ar_), 117.1 (*CH*_Ar_), 111.2 (2× *CH*_Ar_), 109.4 (*C*_Ar_), 55.0 (N*C*H_2_), 52.1 (NH*C*H), 35.9 (*C*H_3_), 30.7 (*C*H_2_COOH), 26.4 (*C*H_2_). HRMS: (ESI^+^-MS, *m*/*z*) calcd for C_22_H_25_N_6_O_5_ [M+H]^+^: 453.1881, found: 453.1862.

#### 3.1.5. Compound **14**: Di-*tert*-butyl (4-((3-cyano-4-fluorobenzyl)thio)benzoyl)-*L*-glutamate

To a stirred solution of **13** [20] (143 mg, 1.00 equiv., 0.18 mmol,) in absolute EtOH (5 mL) under Ar was added NaBH_4_ (18 mg, 2.5 equiv., 0.467 mmol). The suspension was stirred at room temperature for 1 h (complete reduction of the disulfide monitored by TLC (cyclohexane:EtOAc, 6:4). Then, the solvent was evaporated under reduced pressure and the crude mixture was dissolved in anhydrous DMF (3.5 mL). **9** (200 mg, 5 equiv., 0.934 mmol) was added in portions and the reaction was stirred at room temperature for 1 day. The solvent was then evaporated under reduced pressure and the crude was purified by column chromatography (cyclohexane:EtOAc, 8:2) to afford **14 **(164 mg, 0.154 mmol, 86%) as a colorless oil.

R*f *0.60 (cyclohexane:EtOAc, 6:4); ^1^H NMR (300 MHz, CDCl_3_) δ ppm: 7.72 (d, *J* = 8.4 Hz, 2H, 2× C*H*_Ar_), 7.59 (dd, *J* = 5.9, 2.3 Hz, 1H, C*H*_Ar_), 7.56–7.45 (m, 1H, C*H*_Ar_), 7.28 (d, *J* = 8.4 Hz, 2H, 2× C*H*_Ar_), 7.13 (t, *J* = 8.6 Hz, 1H, C*H*_Ar_), 7.01 (d, *J* = 7.3 Hz, 1H, NH), 4.63 (td, *J* = 7.7, 4.5 Hz, 1H, NHC*H*), 4.13 (s, 2H, SC*H*_2_), 2.45–2.17 (m, 3H, *CH*_2_CO + C*H*H), 2.10–1.98 (m, 1H, C*H*H), 1.48 (s, 9H, C(*CH*_3_)_3_), 1.41 (s, 9H, C(*CH*_3_)_3_). ^13^C NMR (75 MHz, CDCl_3_) δ ppm: 172.9 (*C*OO*t*Bu), 171.3 (*C*OO*t*Bu), 166.3 (*C*ONH), 164.2 (CF), 160.7 (O*C*_Ar_), 139.8 (*C*_Ar_), 135.4 (*CH*_Ar_, d, *J *= 8.25 Hz), 134.4 (*C*_Ar_), 133.6 (*CH*_Ar_), 132.2 (*C*_Ar_), 128.9 (2× *CH*_Ar_), 127.9 (2× *CH*_Ar_), 116.8 (*CH*_Ar_, d, *J *= 19.5 Hz), 113.7 (*CH_Ar_*CN), 101.8 (CH_Ar_*CN*), 82.7 (*C*(CH_3_)_3_), 81.1 (*C*(CH_3_)_3_), 53.1 (NH*CH*), 36.9 (*CH*_2_S), 31.8 (CH_2_CO), 28.2 (C(*CH*_3_)_3_), 27.5 (CH_2_). HRMS: (ESI^+^-MS, *m*/*z*) calcd for C_28_H_33_FN_2_NaO_5_S [M+Na]^+^: 551.1986, found: 551.1962.

#### 3.1.6. Compound **15**: Di-*tert*-butyl (4-(((2,4-diaminoquinazolin-6-yl)methyl)thio)benzoyl)-*L*-glutamate

Compound** 14 **(164 mg, 1.00 equiv., 0.31 mmol) and guanidine carbonate (123 mg, 4.4 equiv., 1.365 mmol) were dissolved in anhydrous DMF (7.5 mL) and the mixture was heated at 150 °C for 16 h under Ar. The solvent was evaporated under reduced pressure and the obtained residue was purified by column chromatography (CH_2_Cl_2_:MeOH, 10:0 to 8:2) to provide **15** (110 mg, 0.20 mmol, 63%) as a yellow oil.

R*f *0.40 (CH_2_Cl_2_:MeOH, 85:15); ^1^H NMR (360 MHz, DMSO-d_6_) δ ppm: 8.53 (d, *J* = 7.5 Hz, 1H, CO*NH*), 8.01 (s, 1H, C*H*_Ar_), 7.75 (d, *J* = 8.3 Hz, 2H, 2× C*H*_Ar_), 7.57 (d, *J* = 7.5 Hz, 1H, C*H*_Ar_), 7.46 (bs, 2H, NH_2_), 7.39 (d, *J* = 8.4 Hz, 2H, 2× C*H*_Ar_), 7.17 (d, *J* = 6.3 Hz, 1H, C*H*_Ar_), 6.14 (bs, 1H, NH_2_), 4.30 (m, 3H, NHC*H*, SC*H*_2_), 2.29 (t, *J* = 7.3 Hz, 2H, CH_2_CO), 2.03–1.84 (m, 2H, CH_2_), 1.37 (s, 9H, C(*CH*_3_)_3_), 1.34 (s, 9H, C(*CH*_3_)_3_). ^13^C NMR (91 MHz, DMSO-d_6_) δ ppm: 172.0 (*C*OO*t*Bu), 171.5 (*C*OO*t*Bu), 166.8 (*C*ONH), 166.7 (*C*_Ar_), 162.7 (*C*_Ar_), 160.2 (*C*_Ar_), 142.9 (*C*_Ar_), 141.3 (*C*_Ar_), 134.3 (*CH*_Ar_), 132.7 (*C*_Ar_), 131.0 (*C*_Ar_), 129.4 (*C*_Ar_), 128.4 (2x*CH*_Ar_), 127.0 (2x*CH*_Ar_), 125.8 (*CH*_Ar_), 124.2 (*CH*_Ar_), 81.2 (*C*(CH_3_)_3_), 80.4 (*C*(CH_3_)_3_), 52.9 (NH*CH*), 36.2 (*CH*_2_S), 31.7 (CH_2_CO), 28.1 (C(*CH*_3_)_3_), 28.0 (C(*CH*_3_)_3_), 26.2 (CH_2_). HRMS: (ESI^+^-MS, *m*/*z*) calcd for C_29_H_38_N_5_O_5_S [M+H]^+^: 568.2588, found: 568.2577.

#### 3.1.7. Compound **6**: (4-(((2,4-Diaminoquinazolin-6-yl)methyl)thio)benzoyl)-*L*-glutamic Acid TFA Salt

To a stirred solution of **15** (98 mg, 1.00 equiv., 0.17 mmol) in CHCl_3_ (6 mL) under Ar at room temperature was added TFA (2.4 mL, 180 equiv., 31 mmol) dropwise and the reaction mixture was stirred at room temperature for 16 h. Then, the solvent was removed under reduced pressure, co-evaporated twice with MeOH (25 mL) and the brown residue was triturated with Et_2_O (5 mL) to provide a white solid. The crude product was filtered with Et_2_O (30 mL) and acetone (30 mL) to afford **6**.CF_3_COOH (71 mg, 0.125 mmol, 73%) as off-white solid.

mp > 260 °C; ^1^H NMR (360 MHz, DMSO-d_6_) δ ppm: 8.34 (d, *J* = 7.6 Hz, 1H, CO*NH*), 8.10 (s, 1H, C*H*_Ar_), 7.80 (d, *J* = 8.6 Hz, 4H, 2× C*H*_Ar_* + NH*_2_), 7.61 (d, *J* = 8.6 Hz, 1H, C*H*_Ar_), 7.41 (d, *J* = 8.4 Hz, 2H, 2× C*H*_Ar_), 7.21 (d, *J* = 8.5 Hz, 1H, C*H*_Ar_), 6.82 (s, 2H, *NH*_2_), 4.32 (m, 3H, SC*H*_2 _*+ *NHC*H*), 2.30 (t, *J* = 7.3 Hz, 2H, C*H*_2_COO), 2.10–1.90 (m, 2H, C*H*_2_). ^13^C NMR (91 MHz, DMSO-d_6_) δ ppm: 174.5 (*C*OOH), 174.4 (*C*OOH), 165.5 (*C*OHN), 162.4 (*C*_Ar_), 158.8 (*C*_Ar_), 158.3 (*C*_Ar_), 140.5 (*C*_Ar_), 134.3 (*CH*_Ar_), 131.2 (*C*_Ar_), 127.9 (2× *CH*_Ar_), 126.7 (2× *CH*_Ar_), 125.4 (*CH*_Ar_), 124.2 (*CH*_Ar_), 109.6 (*C*_Ar_), 52.7 (NH*C*H), 35.9 (S*C*H_2_), 31.0 (*C*H_2_COOH), 26.8 (*C*H_2_). MS ESI+: [M+H]^+^ 456.1, [M+Na]^+^ 478.1. HRMS: (ESI^+^-MS, *m*/*z*) calcd for C_21_H_22_N_5_O_5_S [M+H]^+^: 456.1336, found: 456.1321.

#### 3.1.8. Compound **17**: Di-*tert*-butyl (4-((3-cyano-4-fluorobenzyl)oxy)benzoyl)-*L*-glutamate

Compound **16 [20]** (532 mg, 2.00 equiv., 1.40 mmol) and **9** (150 mg, 1.00 equiv., 0.700 mmol) were dissolved in anhydrous DMF (1.75 mL). Potassium carbonate (388 mg, 4 equiv., 2.80 mmol) was added and the mixture was stirred at room temperature under Ar for 1 day. The mixture was then diluted with EtOAc, followed by washing with water and brine. The organic phase was dried over MgSO_4_, filtered, and evaporated under reduced pressure. The residue was purified by flash column chromatography (cyclohexane:EtOAc, 8:2) to give **17** (334 mg, 1.30 mmol, 93%) as a colorless oil.

R*f *0.45 (cyclohexane:EtOAc, 6:4); ^1^H NMR (300 MHz, CDCl_3_) δ ppm: 7.83–7.79 (m, 2H, 2× C*H*_Ar_), 7.74–7.65 (m, 2H, 2× C*H*_Ar_), 7.25 (t, *J* = 8.9 Hz, 1H, CFC*H*_Ar_), 6.99–6.95 (m, 2H, 2× C*H*_Ar_), 6.93 (d, *J* = 9.0 Hz, 1H, NH), 5.09 (s, 2H, C_Ar_C*H*_2_), 4.65 (td, *J* = 7.8, 4.6 Hz, 1H, NHC*H*), 3.09 (s, 3H, C*H*_3_), 2.48–2.17 (m, 3H, C*H*HCH_2_CO, CH_2_CO), 2.17–1.98 (m, 1H, C*H*HCH_2_CO), 1.49 (s, 9H, C(*CH*_3_)_3_), 1.41 (s, 9H, C(*CH*_3_)_3_). ^13^C NMR (75 MHz, CDCl_3_) δ ppm: 172.8 (*C*OO*t*Bu), 171.5 (*C*OO*t*Bu), 166.4 (*C*ONH), 164.6 (CF), 161.2 (O*C*_Ar_), 160.8 (CF), 134.0 (*CH*_Ar_, d, *J *= 7.95 Hz), 133.8 (*C*_Ar_), 132.4 (*CH*_Ar_), 129.3 (2× *CH*_Ar_), 127.4 (CO*C*_Ar_), 117.0 (*CH*_Ar_, d, *J *= 20.25 Hz), 114.6 (2× *CH*_Ar_), 113.8 (*CH_Ar_*CN), 102.1 (CH_Ar_*CN*), 82.6 (*C*(CH_3_)_3_), 81.0 (*C*(CH_3_)_3_), 68.3 (*CH*_2_C_Ar_), 53.0 (NH*CH*), 31.8 (CH_2_CO), 28.2 (C(*CH*_3_)_3_), 27.7 (CH_2_). HRMS: (ESI^+^-MS, *m*/*z*) calcd for C_28_H_33_FN_2_NaO_6_ [M+H]^+^: 535.2215, found: 535.2194.

#### 3.1.9. Compound **18**: Di-*tert*-butyl (4-((2,4-diaminoquinazolin-6-yl)methoxy)benzoyl)-*L*-glutamate

Compound **17 **(232 mg, 1.00 equiv., 0.45 mmol) and guanidine carbonate (57 mg, 1.40 equiv., 0.634 mmol) were dissolved in anhydrous DMF (10 mL) and the mixture was heated at 150 °C for 4 h under Ar. The solvent was evaporated under reduced pressure and the obtained residue was purified by column chromatography (CH_2_Cl_2_:MeOH, 10:0 to 8:2) to provide **18** (114 mg, 0.21 mmol, 46%) as a white glassy solid.

R*f *0.55 (CH_2_Cl_2_:MeOH, 85:15); mp 116 °C; ^1^H NMR (300 MHz, DMSO-d_6_) δ ppm: 8.42 (d, *J* = 7.6 Hz, 1H, CO*NH*), 8.12 (d, *J* = 1.3 Hz, 1H, C*H*_Ar_), 7.87 (d, *J* = 8.8 Hz, 2H, 2× C*H*_Ar_), 7.59 (dd, *J* = 8.6, 1.7 Hz, 1H, C*H*_Ar_), 7.39 (bs, 2H, NH_2_), 7.22 (d, *J* = 8.6 Hz, 1H, C*H*_Ar_), 7.12 (d, *J* = 8.9 Hz, 2H, 2× C*H*_Ar_), 6.12 (bs, 2H, NH_2_), 5.12 (s, 2H, OC*H*_2_), 4.31 (dd, *J* = 12.7, 9.6 Hz, 1H, NHC*H*), 2.33 (t, *J* = 7.5 Hz, 2H, CH_2_CO), 2.07–1.87 (m, 2H, C*H*_2_), 1.41 (s, 9H, C(*CH*_3_)_3_), 1.38 (s, 9H, C(*CH*_3_)_3_). ^13^C NMR (75 MHz, DMF-d_7_) δ ppm: 172.2 (*C*OO*t*Bu), 171.8 (*C*OO*t*Bu), 167.7 (*C*ONH), 166.7 (*C*_Ar_), 163.6 (*C*_Ar_), 161.7 (*C*_Ar_), 160.6 (*C*_Ar_), 133.5 (*CH*_Ar_), 129.9 (*C*_Ar_), 129.6 (*CH*_Ar_), 127.2 (*C*_Ar_), 124.1 (*CH*_Ar_), 123.3 (*CH*_Ar_), 114.7 (*CH*_Ar_), 110.4 (*C*_Ar_), 80.9 (*C*(CH_3_)_3_), 80.1 (*C*(CH_3_)_3_), 70.2 (*CH*_2_O), 53.3 (NH*CH*), 31.9 (CH_2_CO), 27.7 (C(*CH*_3_)_3_), 27.7 (C(*CH*_3_)_3_), 26.8 (CH_2_). MS ESI^+^: [M+H]^+^ 552.3, [M+Na]^+^ 574.3. HRMS: (ESI^+^-MS, *m*/*z*) calcd for C_29_H_38_N_5_O_6_ [M+H]^+^: 552.2817, found: 552.2799.

#### 3.1.10. Compound **7**: (4-((2,4-Diaminoquinazolin-6-yl)methoxy)benzoyl)-*L*-glutamic Acid TFA Salt

To a well stirred solution of **18** (110 mg, 1.00 equiv., 0.199 mmol) in CHCl_3_ (7 mL) under Ar at room temperature, TFA was added (2.8 mL, 180 equiv., 35.9 mmol) dropwise and the reaction mixture was stirred at room temperature for 16 h. Then, the solvent was removed under reduced pressure and the brown residue was triturated with Et_2_O (5 mL) to provide a white solid. The crude product was washed with Et_2_O (30 mL) and acetone (30 mL) to afford **7**.CF_3_COOH (86 mg, 0.155 mmol, 78%) as white solid.

mp > 260 °C; ^1^H NMR (300 MHz, DMSO-d_6_) δ ppm: 8.91 (d, *J* = 55.1 Hz, 2H, *NH*_2_), 8.48 (d, *J* = 7.2 Hz, 1H, CO*NH*), 8.38 (s, 1H, C*H*_Ar_), 8.12 (s, 2H, *NH*_2_), 7.89 (d, *J* = 8.1 Hz, 3H, 3× C*H*_Ar_), 7.45 (d, *J* = 8.2 Hz, 1H, C*H*_Ar_), 7.13 (d, *J* = 8.1 Hz, 2H, 2× C*H*_Ar_), 5.21 (s, 2H, OC*H*_2_), 4.38 (s, 1H, NHC*H*), 2.35 (t, *J* = 7.2 Hz, 2H, C*H*_2_COO), 2.12–1.91 (m, 2H, C*H*_2_). ^13^C NMR (75 MHz, DMSO-d_6_) δ ppm: 174.0 (*C*OOH), 173.7 (*C*OOH), 166.0 (*C*OHN), 163.1 (*C*_Ar_), 160.6 (*C*_Ar_), 158.9 (*C*_Ar_), 158.5 (*C*_Ar_), 155.0 (*C*_Ar_), 139.4 (*C*_Ar_), 135.3 (*CH*_Ar_), 132.6 (*C*_Ar_), 129.4 (*CH*_Ar_), 126.6 (*C*_Ar_), 124.4 (*CH*_Ar_), 117.1 (*CH*_Ar_), 114.4 (*CH*_Ar_), 109.3 (*C*_Ar_), 68.9 (O*C*H_2_), 52.0 (NH*C*H), 30.5 (*C*H_2_COOH), 26.0 (*C*H_2_). MS ESI+: [M+H]^+^ 440.2, [M+Na]^+^ 462.1. HRMS: (ESI^+^-MS, *m*/*z*) calcd for C_21_H_22_N_5_O_6_ [M+H]^+^: 440.1565, found: 440.1546.

### 3.2. Cytotoxicity Experiments on A549 Human Cancer Cells

A549 cells were cultured in DMEM (Dulbecco’s Modified Eagle Medium) supplemented with 10% of fetal calf serum (FCS) and 100 U/mL penicillin and 100 µg/mL streptomycin in a 5% CO_2_/95% hygrometry environment at 37 °C.

The cytotoxic activity of methotrexate and analogs has been determined using the MTS ((3-(4,5-dimethylthiazol-2-yl)-5-(3-carboxymethoxyphenyl)-2-(4-sulfophenyl)-2*H*-tetrazolium)) method (Promega). A total of 2.5 × 10^3^ cells (100 µL) were seeded in 96-well plates and incubated overnight at 37 °C in the presence of 5% CO_2_ and 95% hygrometry. Then, 100 µL of each compound (**1**, **3**, **6**, and **7**) were added at different concentrations. After 72 h, the media was removed and 100 µL of MTS solution (10% in media) were added in each well. After a 3 h incubation period at 37 °C (5% CO_2_ and 95% hygrometry), the optical density was measured at 490 nm wavelength using a microplate reader (Infinite M200 Pro, Tecan trading AG, Männedorf, Switzerland). Untreated cells were used as control. Each concentration was tested in six replicates, and the experiment was carried out in triplicates. The concentration inhibiting 50% of the cell proliferation (IC_50_) was calculated using GraphPad Prism software version 8.0.2 (GraphPad Software Inc., San Diego, CA, USA).

### 3.3. DHFR Inhibition Assay

The Dihydrofolate Reductase Assay Kit was obtained from Sigma-Aldrich (St. Louis, MO, USA). The assay system contained 0.1 units of DHFR in phosphate buffer (pH 7.5, concentration is estimated to be 0.32 µM–0.96 µM), Assay Buffer 10X for DHFR, Dihydrofolic acid substrate, Methotrexate, and NADPH. The dihydrofolic acid substrate, NADPH, methotrexate, and the other 3 inhibitors tested (**1**, **3**, **6** and **7** in 1% DMSO solution) were prepared as a 10 mM stock solution at pH 7.5 and aliquoted. The enzyme inhibition was tested in a mixture of assay buffer, 15 µL of DHFR enzyme, 6 µL NADPH, 5 µL dihydrofolic acid substrate, and 10 µL of the inhibitor at various concentration. The spectrophotometer was set at 340 nm, at 22 °C and in the kinetics program mode. The absorbance was measured at this wavelength every 15 s for 2.5 min. The inhibitor concentration necessary to reduce enzyme activity to half the uninhibited value was determined using GraphPad Prism software (GraphPad Software Inc., San Diego, CA, USA).

### 3.4. In Vitro Metabolism

#### 3.4.1. Monitoring of Hepatic Metabolism

Analyses were performed on Agilent 1260 Infinity II LC System coupled to an Agilent mass spectrometry MS SQ 6125 (ESI/Q). UV detection was primarily made at 280 nm. Elutions were performed using a Macherey-Nagel Nucleodur C18 Gravity-SB column (3 µm; 4.6 × 75 mm). Mobile phases consisted of solution A, acetonitrile/formic acid 0.1% (*v*/*v*) and solution B, water/formic acid 0.1% (*v*/*v*). The column was eluted with a gradient linearly increasing from 2 to 98% of solution A during 11 min. The flow rate was 0.5 mL/min.

#### 3.4.2. Aldehyde Oxidase Assay

The protocol for aldehyde oxidase purification was carried out as described by Espinosa et al. [20]. Briefly, aldehyde oxidase was partially purified from three Albino rabbit livers (≈300 g, obtained from Pel-Freez Biologicals, Rodgers, AR, USA). Enzymatic activity was verified spectrophotometrically (420 nm, quartz cuvette, Varian Cary 300-bio spectrophotometer, Walnut Creek, CA, USA) at 37 °C. Each solution of methotrexate or derivatives (10 µM) in 10 mM Tris-HCl, pH 7.4 containing 20 mM MgCl_2_ and 1% DMSO (*v*/*v*) were added to a diluted enzyme solution (10 µM) and were incubated at 37 °C for 24 h. Parent compounds and possible metabolite were analyzed by HPLC-MC using the method described above.

#### 3.4.3. Human Liver Cytosol Assay

Methotrexate or derivative solutions (800 µM) were prepared in 10 mM Tris-HCl, pH 7.4 containing 20 mM MgCl_2_ and 1% DMSO (*v*/*v*). A total of 400 µL of compound solution was mixed with 400 µL of pre-warmed human liver cytosol (Thermo Fisher Scientific, Waltham, MA, USA) to a final concentration of 10 mg total protein/mL. The blend was softly shaken in a 37 °C incubator and samples (80 µL) were taken at different time intervals up to 28 h of reaction. Each sample was filtered (PTFE pore size 0.2 μm) before analysis by HPLC-MS.

#### 3.4.4. Human Pooled S9 Fractions Assay

Human pooled S9 fractions (Thermo Fisher Scientific) were diluted 20-fold in PBS buffer (1 mg/mL of proteins). Then, 100 µM and 1 mM solutions of methotrexate or derivatives (150 µL) were incubated for 0 and 12 h at 37 °C. Metabolization was investigated in the presence or absence of 1 mM NADPH and monitored by HPLC-MS.

## 4. Conclusions

Herein, we designed a new synthetic pathway in five steps of the 5,8-dideazamethotrexate, notably, by coupling a building-block to the benzoyl glutamyl structures followed by the formation of the quinazoline moiety. This method was used for the preparation of an ether and a thioether analog of the latter compound, and resulted in quite good overall yields. All analogs showed in vitro cytotoxic activity on the A549 lung cancer cell line and were also potent dihydrofolate reductase inhibitors in the same range. Interestingly, dideaza derivatives of MTX are not hydroxylated by aldehyde oxidase, thus avoiding the formation of potentially toxic metabolites. Moreover, no metabolites were detected in the in vitro tests performed, an interesting result given that high metabolic sensitivity generally results in low bioavailability and high clearance. More specifically, the quinazoline core is a good scaffold for the future development of new methotrexate analogs displaying both better antitumor activity and lower metabolism than the parent compound.

## Figures and Tables

**Table 1:** Effects of methotrexate and analogs on the growth of A549 cancer cells and the inhibition of Dihydrofolate Reductase (DHFR).

Compounds	A549 IC_50_ (µM) ± SD	DHFR IC_50_ (nM) ± SD
Methotrexate (**1**)	0.12 ± 0.02	30 ± 3
5,8-Dideaza-methotrexate (**3**)	0.04 ± 0.01	7 ± 2
** 6 **	0.36 ± 0.04	13 ± 4
** 7 **	0.12 ± 0.02	30 ± 3

## Data Availability

Data are available within the article and Supplementary Materials.

## Supplementary Materials

The following supporting information can be downloaded at: https://www.mdpi.com/article/10.3390/molecules30132772/s1. ^1^H NMR, ^13^C NMR of the compounds and Chromatograms of compounds incubated with human pooled S9 fractions.
