# Supplementary material for: Synthesis and In Vitro Pharmacological Evaluation of 5,8-Dideaza Analogs of Methotrexate
Source: Molecules. 2025 Jun 27;30(13):2772. doi: 10.3390/molecules30132772 (PMC12250909; doi:10.3390/molecules30132772)

Synthesis and in vitro evaluation of 5,8-dideaza analogs of methotrexate

Marta Abellán-Flos, Charles Skarbek, Dáire J. Gibbons, Estelle Rascol, Raphaël Labruère*

Université Paris-Saclay, CNRS, Institut de chimie moléculaire et des matériaux d'Orsay 91405, Orsay, France.

The Supporting Information reports on:

• ^{1}H NMR and ^{13}C NMR spectra of **3**, **6**, **7**, **11**, **12**, **14**, **15**, **17**, **18**

• HRMS spectra of **3**, **6**, **7**

• Chromatograms of compounds **1, 2, 6, 7** incubated for 12 h at 37 °C with human pooled S9 fractions.

^{1}H NMR (300 MHz, $CDCl_3$) - 11

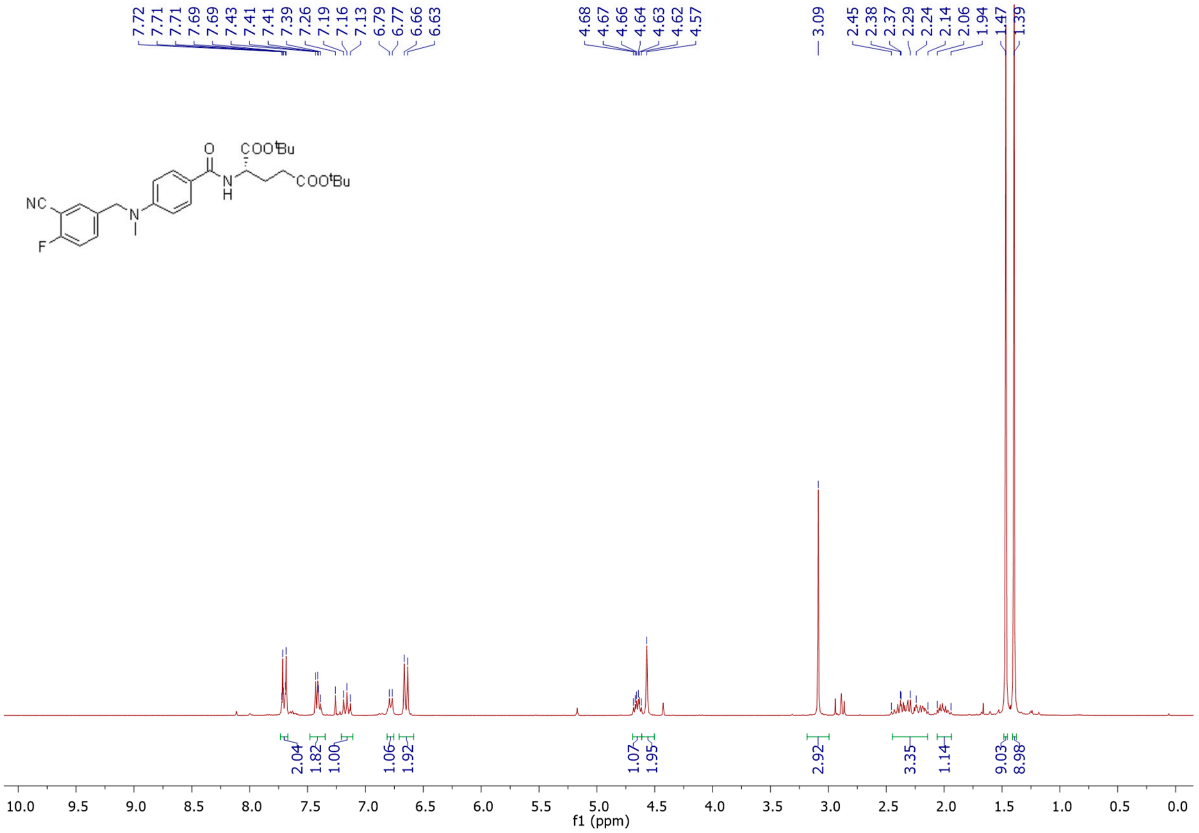

^{13}C NMR (75 MHz, $CDCl_3$) - 11

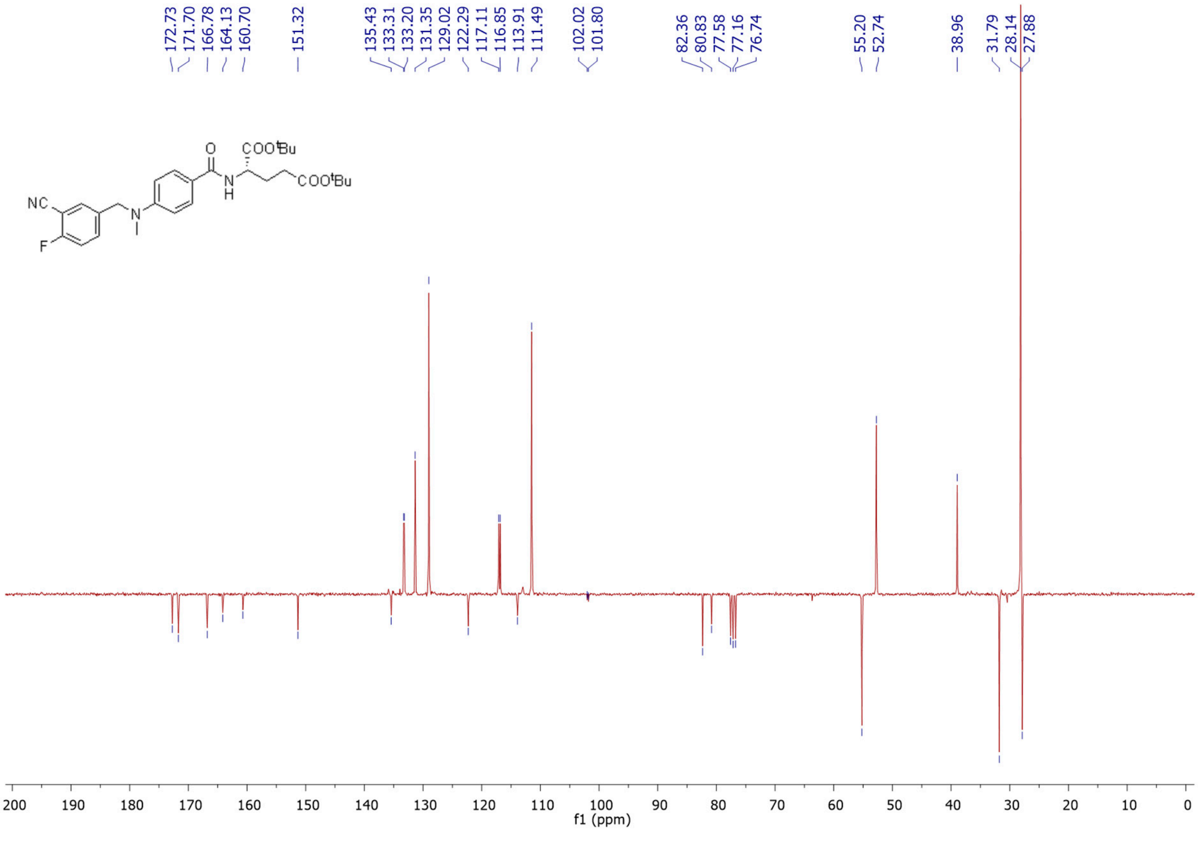

^{1}H NMR (300 MHz, DMSO-d$_6$) - 12

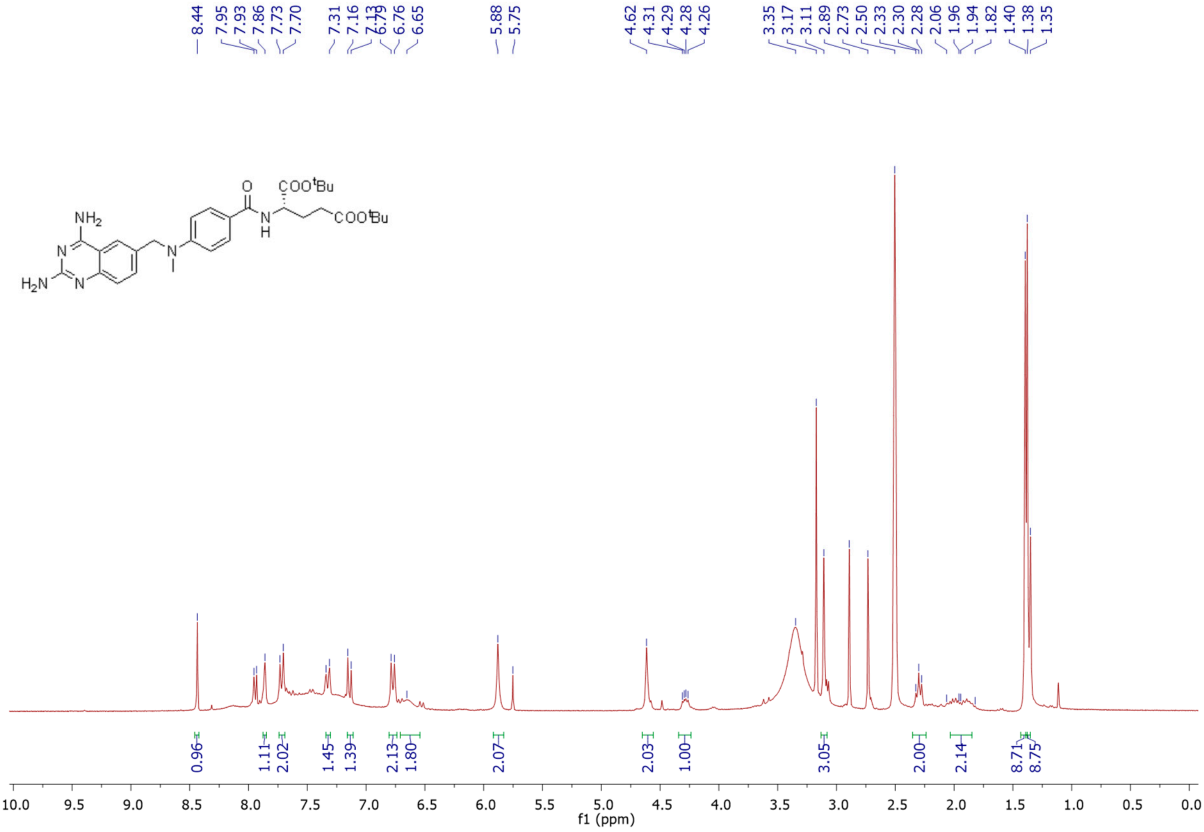

^{13}C NMR (75 MHz, DMSO-d$_6$) - 12

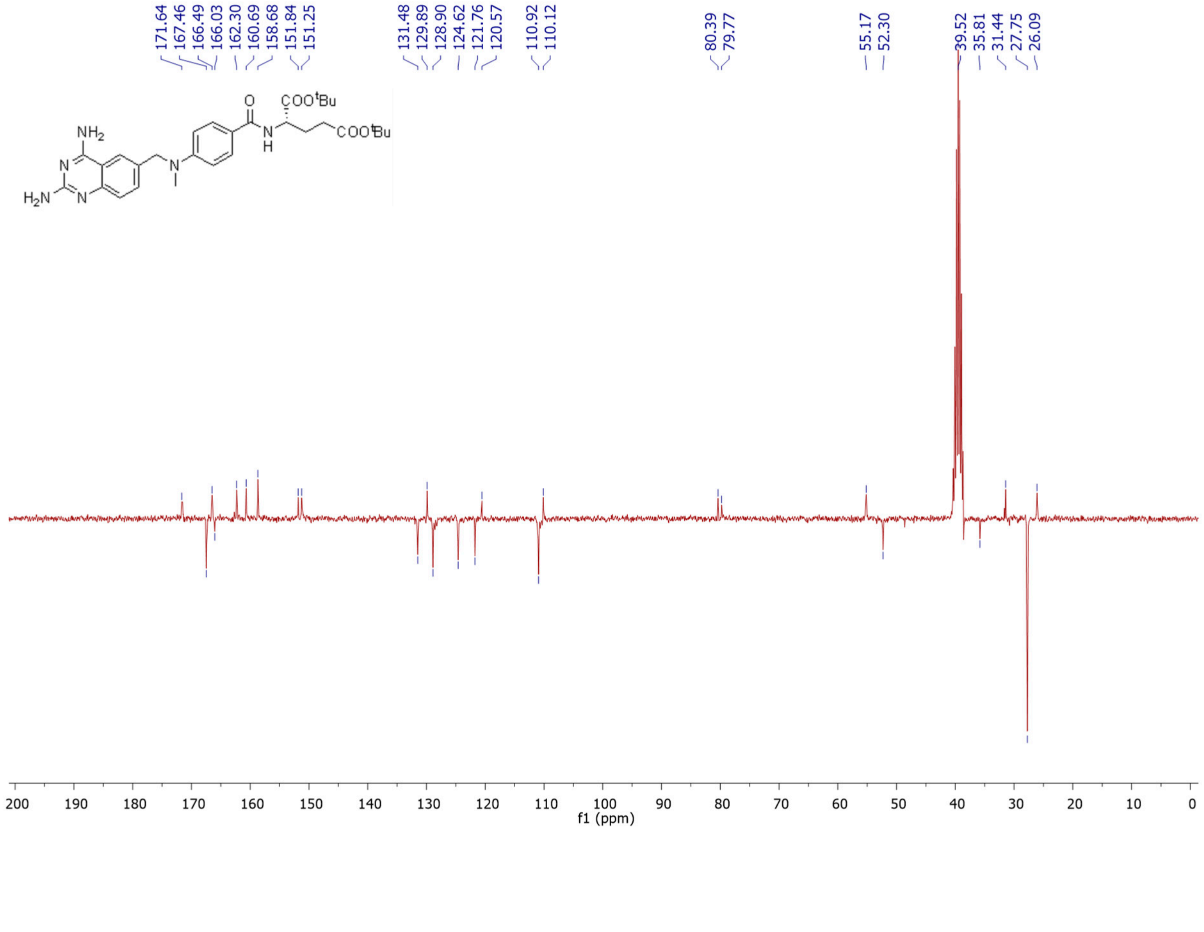

^{1}H NMR (360 MHz, DMSO-d$_6$) - 3

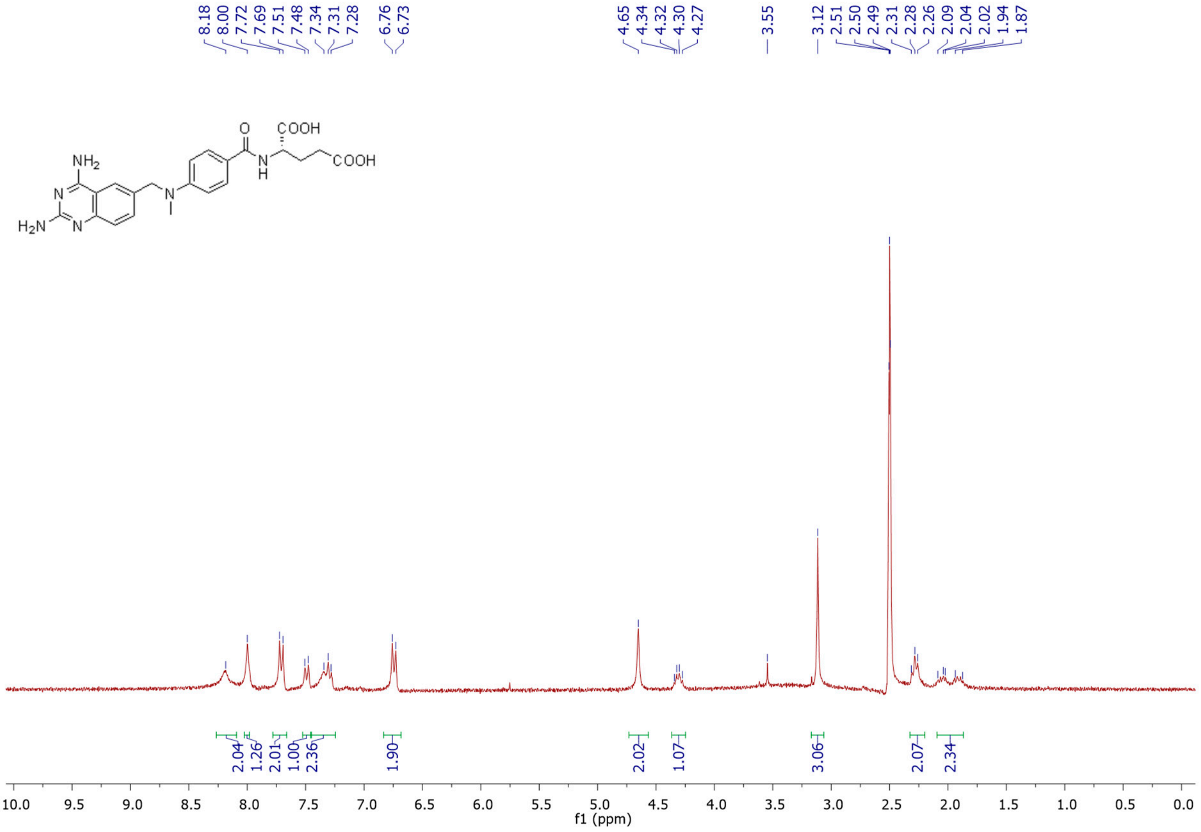

^{13}C NMR (75 MHz, DMSO-d$_6$) - 3

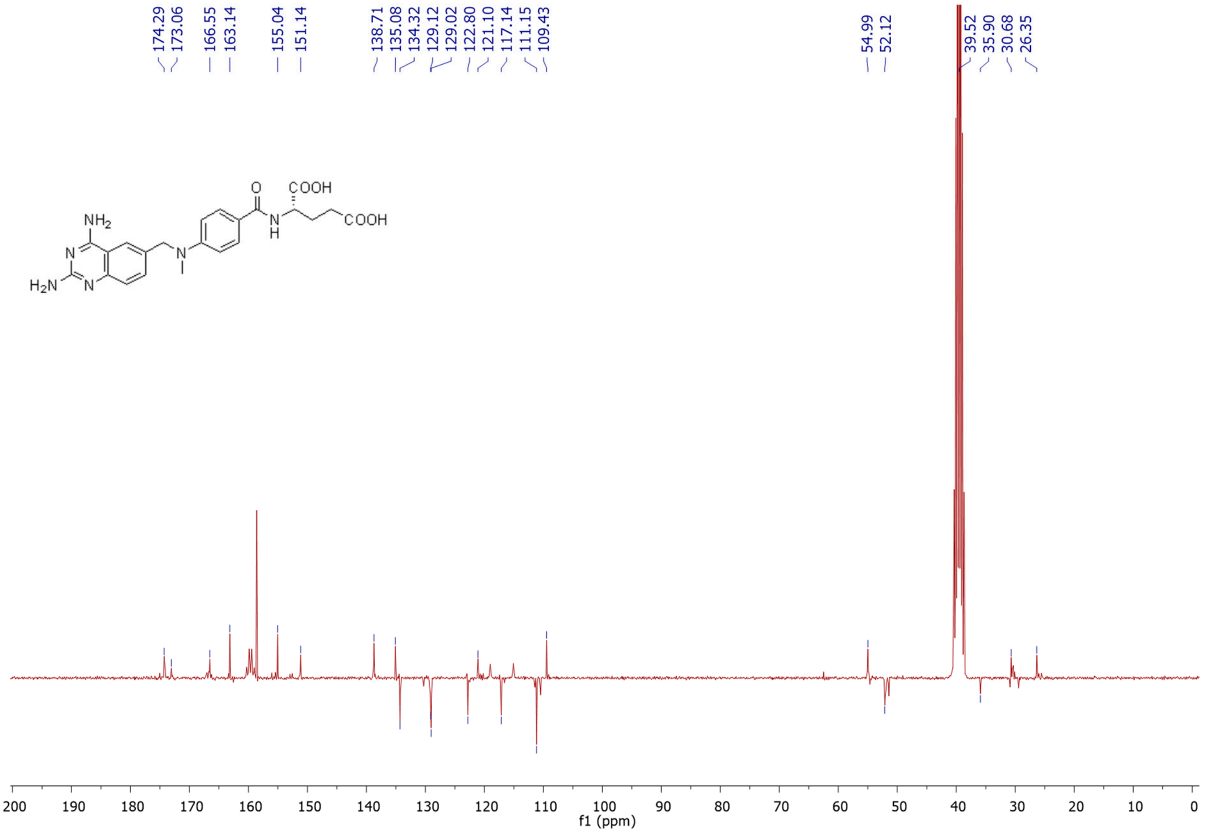

^{1}H NMR (300 MHz, $CDCl_3$) - 17

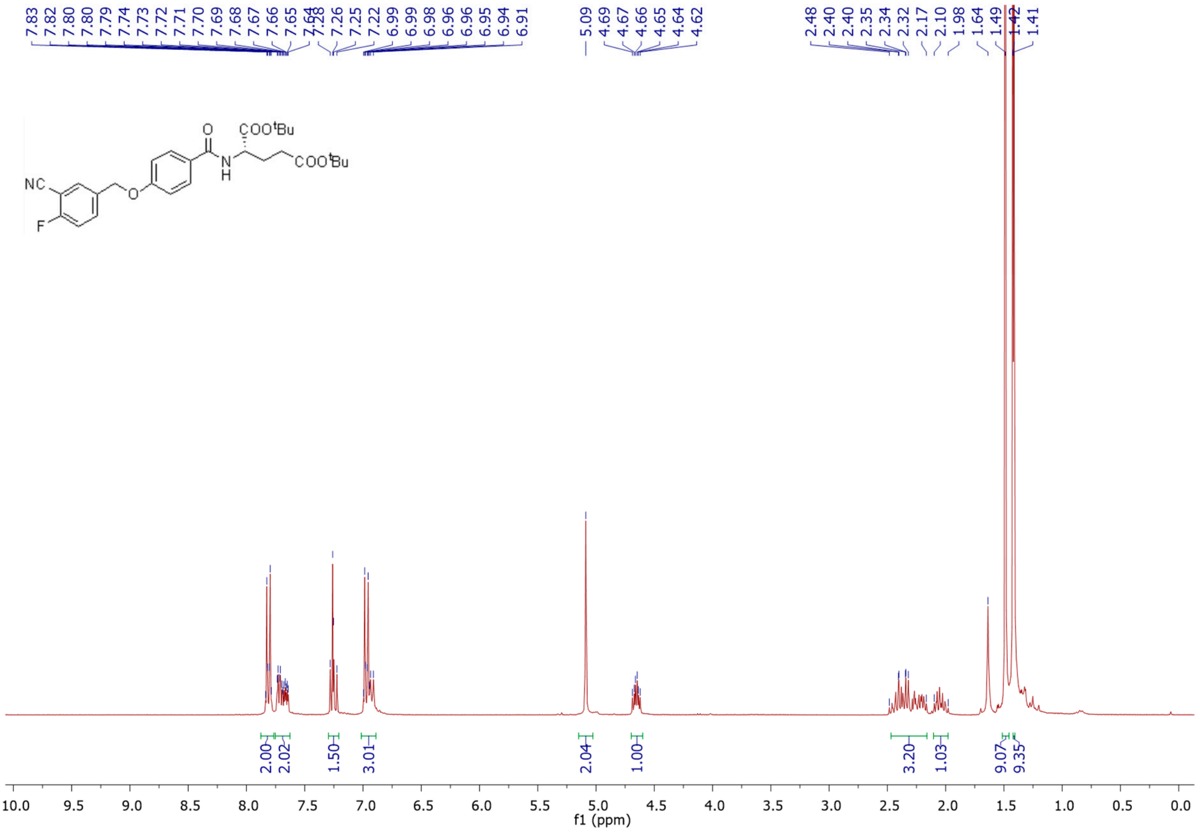

^{13}C NMR (75 MHz, $CDCl_3$) - 17

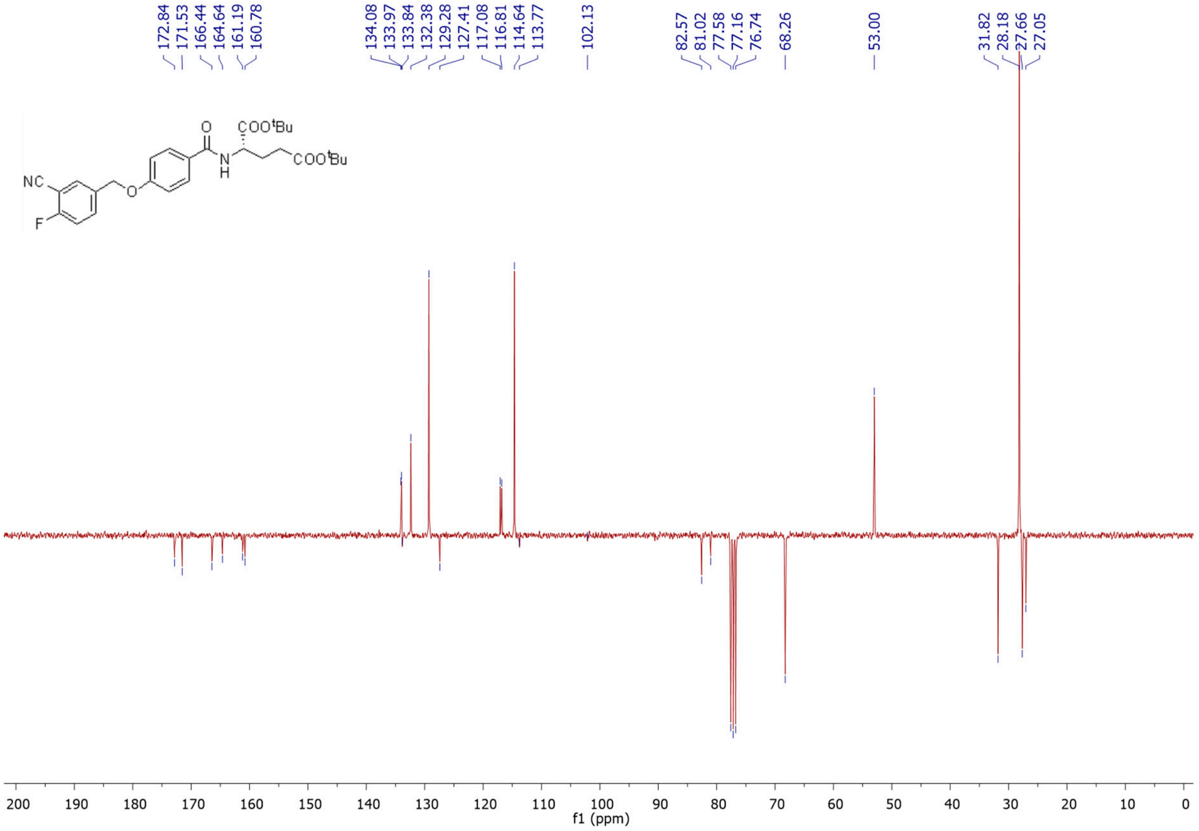

^{1}H NMR (300 MHz, DMSO-d$_6$) - 18

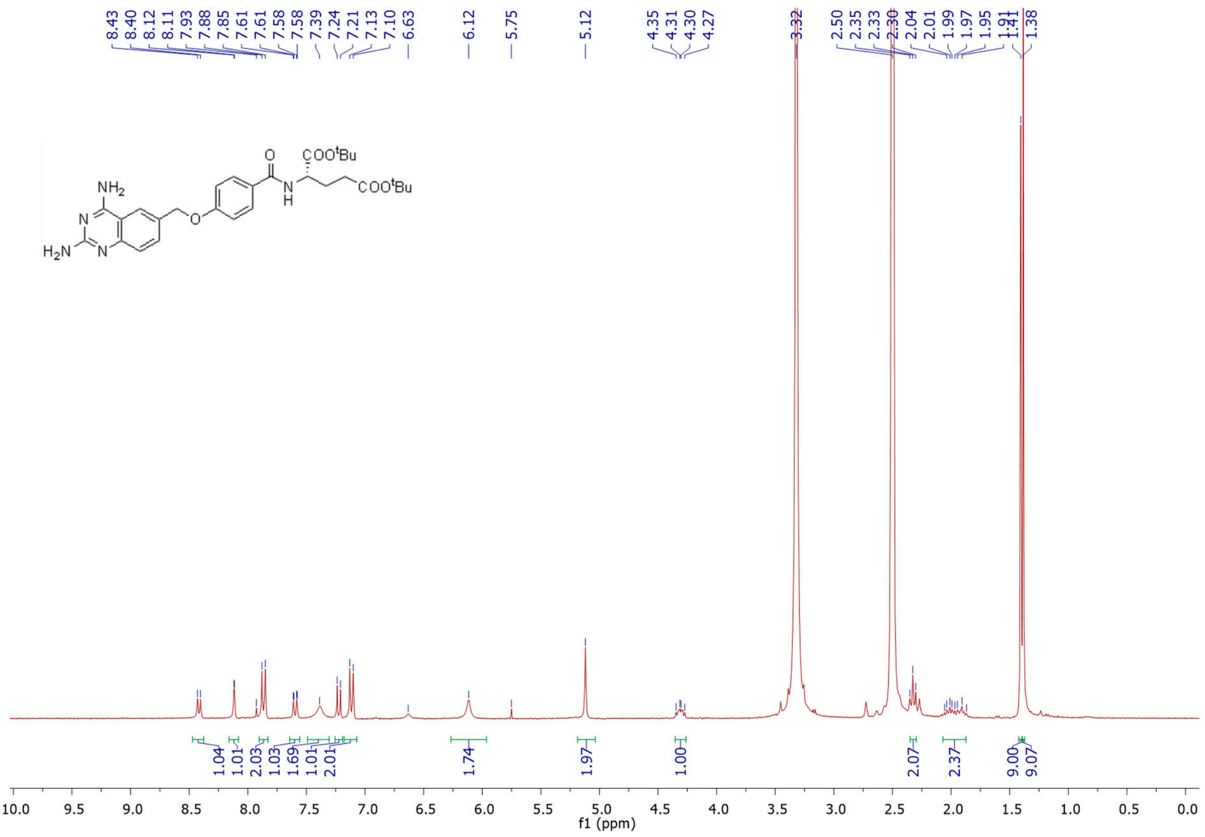

^{13}C NMR (75 MHz, DMF-d$_7$) - 18

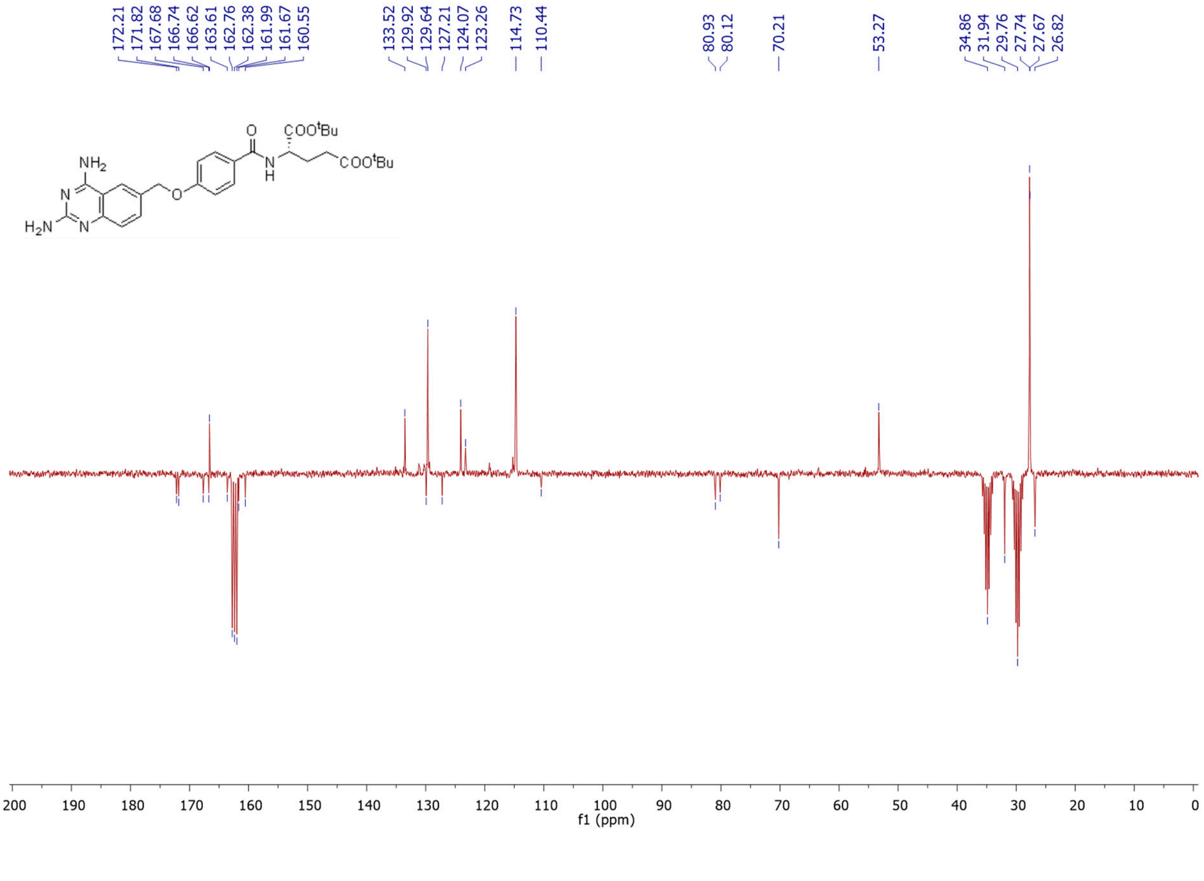

^{1}H NMR (300 MHz, DMSO-d$_6$) - 7

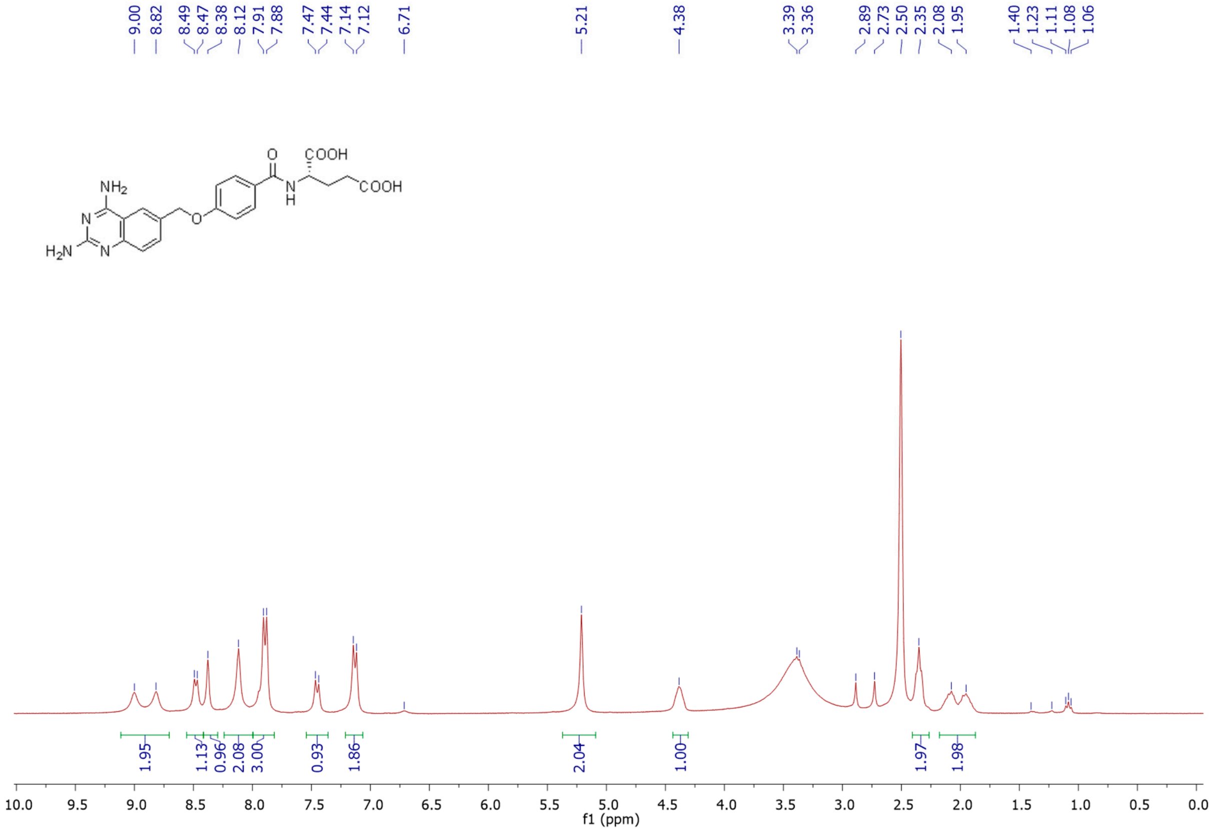

^{13}C NMR (75 MHz, DMSO-d$_6$) - 7

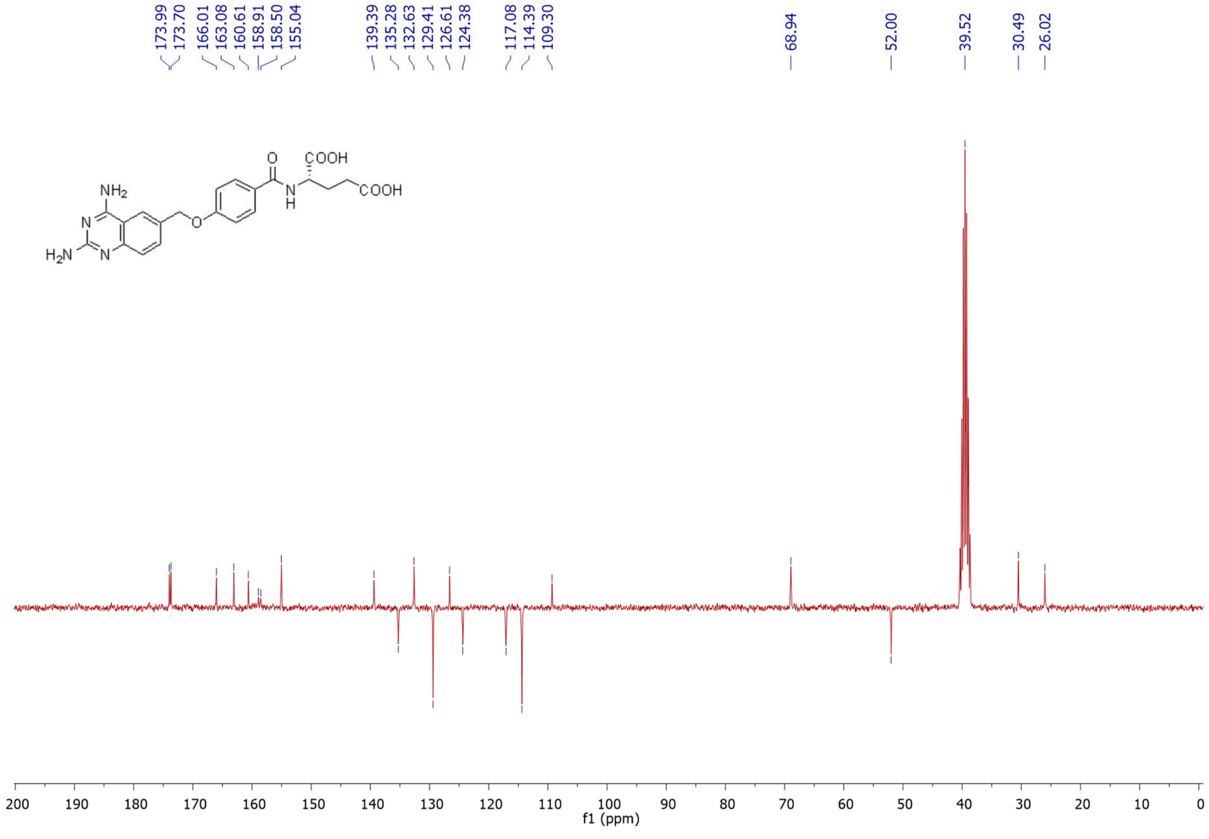

^{1}H NMR (300 MHz, $CDCl_3$) - 14

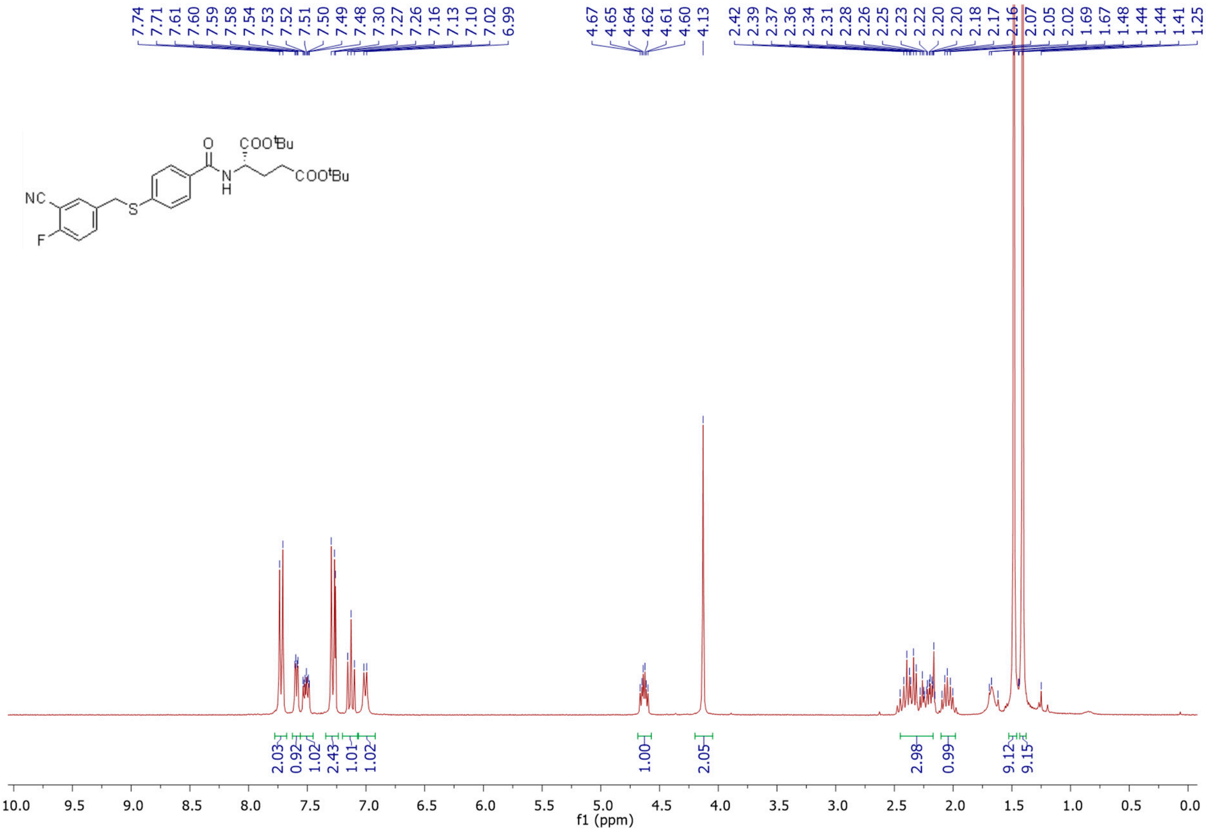

^{13}C NMR (75 MHz, $CDCl_3$) - 14

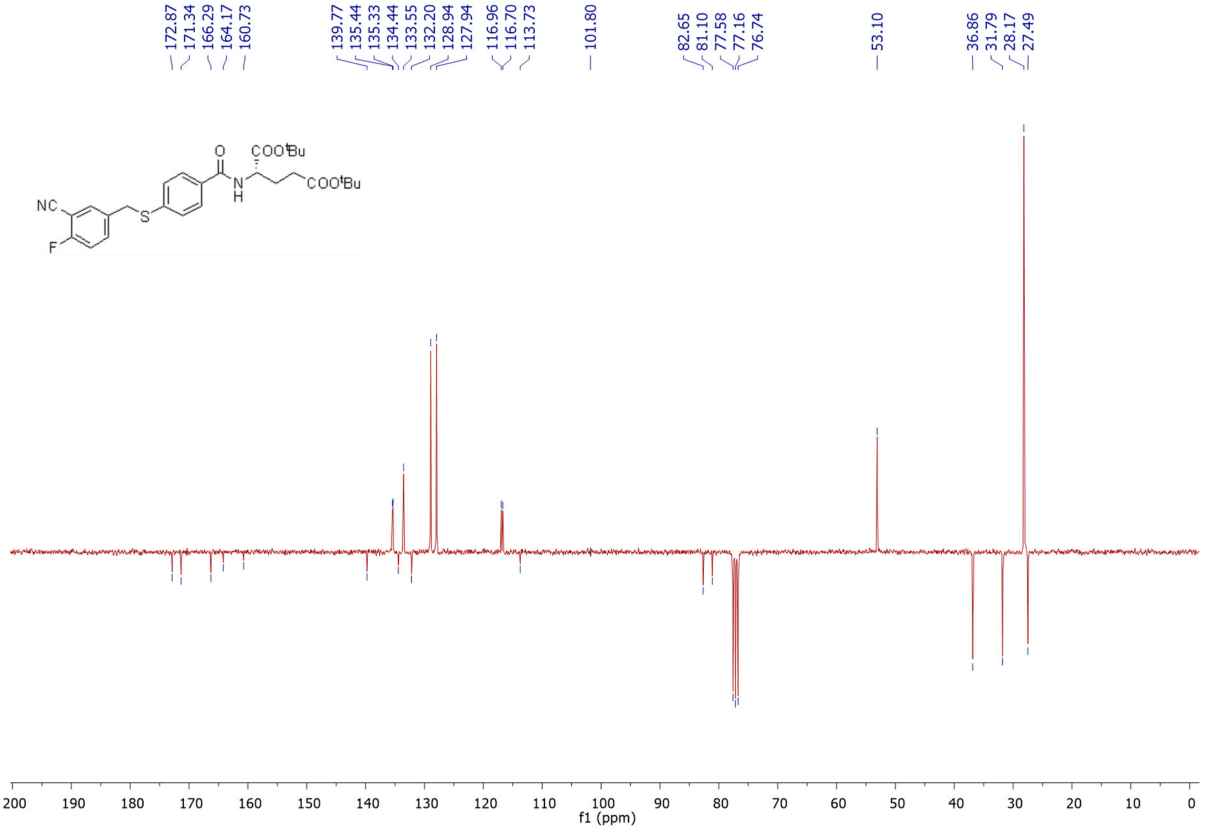

^{1}H NMR (360 MHz, DMSO-d$_6$) - 15

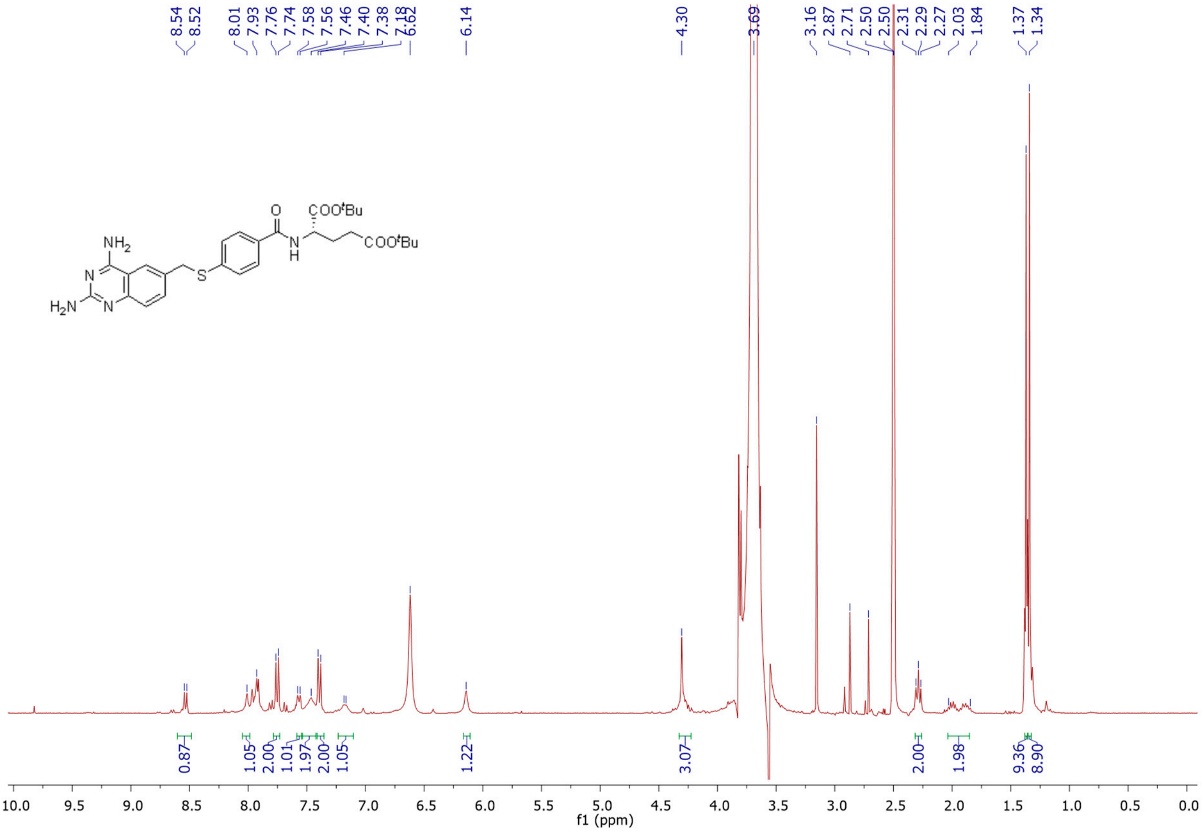

^{13}C NMR (91 MHz, DMSO-d$_6$) - 15

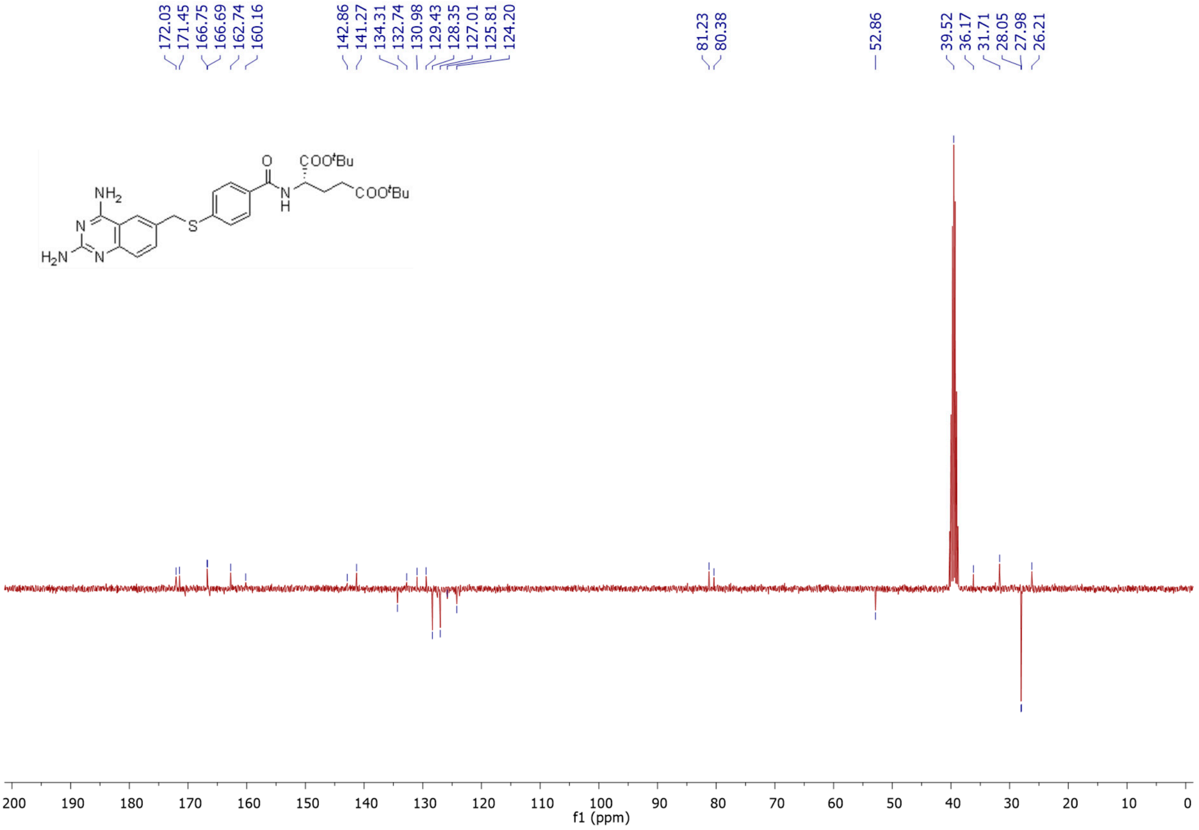

^{1}H NMR (360 MHz, DMSO-d$_6$) - 6

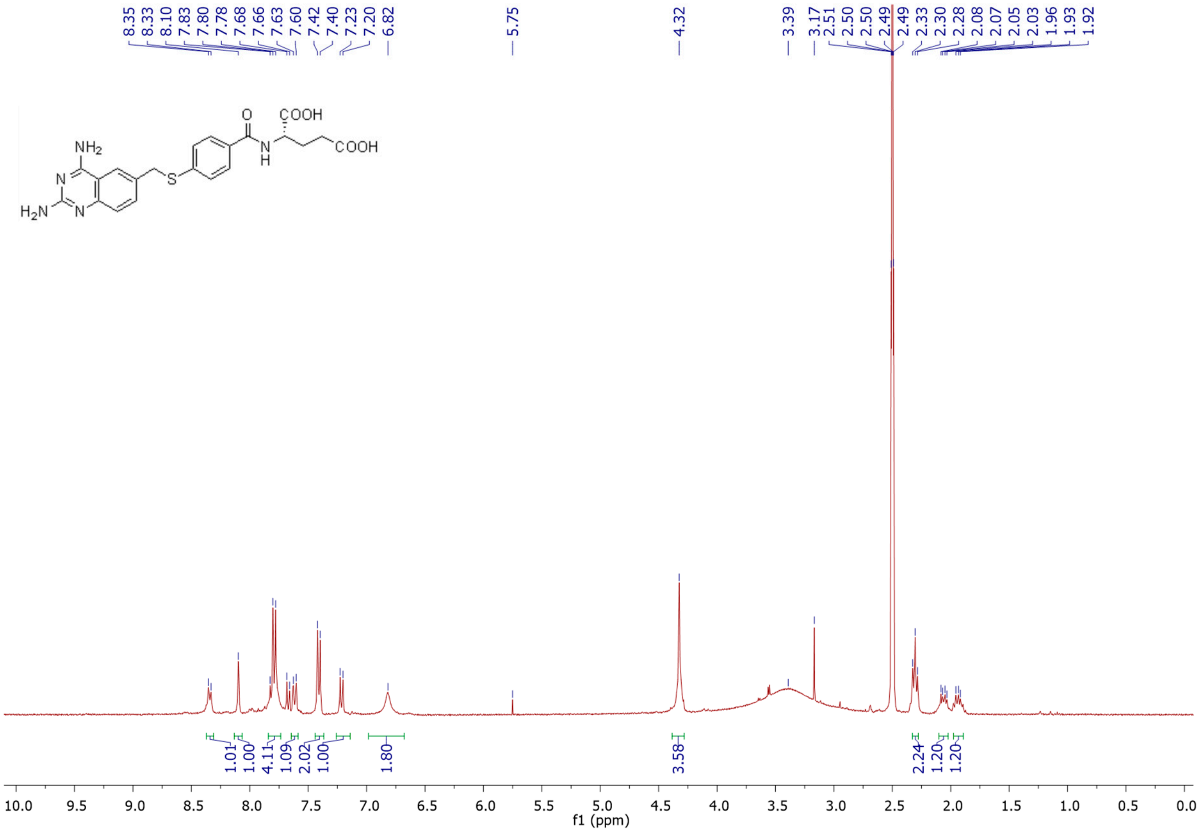

^{13}C NMR (91 MHz, DMSO-d$_6$) - 6

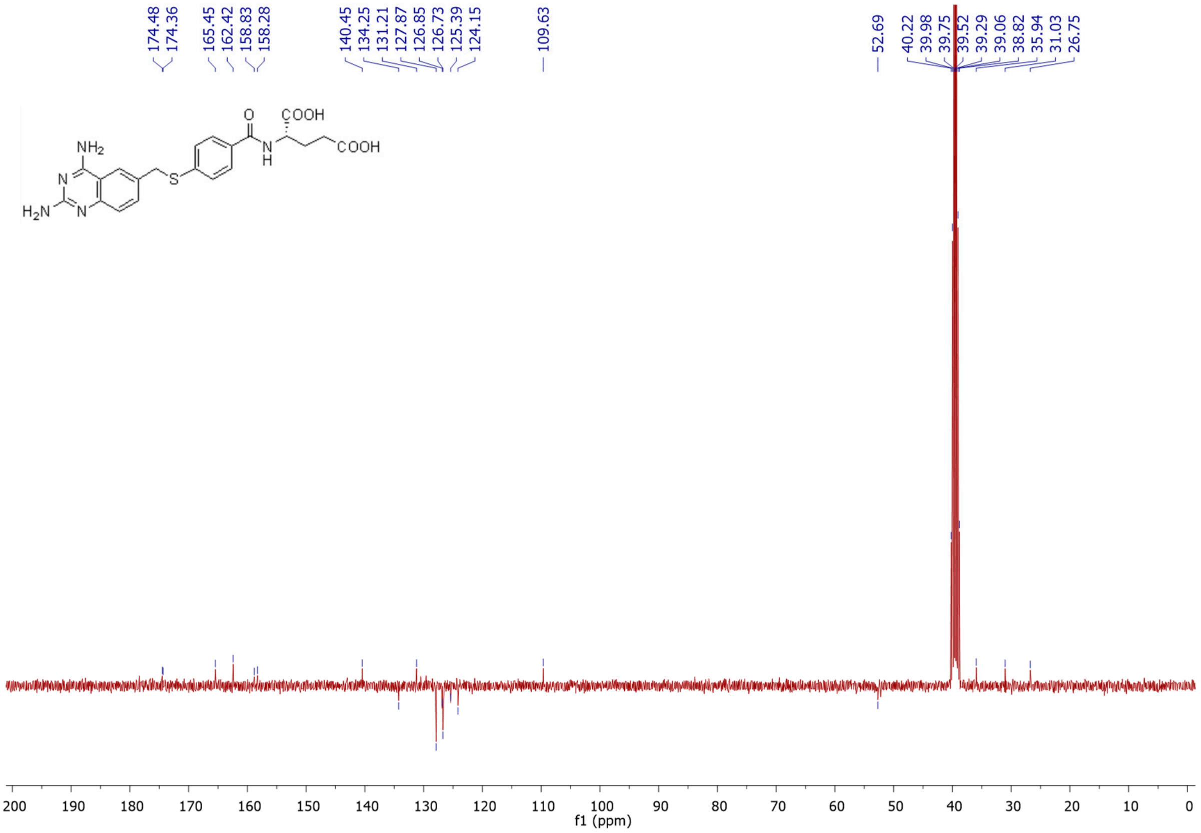

HRMS – 3

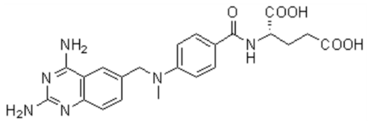

Acquisition Parameter

Source Type	ESI	Ion Polarity	Positive	Set Nebulizer	2.8 Bar
Focus	Not active	Set Capillary	4500 V	Set Dry Heater	180 °C
Scan Begin	50 m/z	Set End Plate Offset	-500 V	Set Dry Gas	9.0 l/min
Scan End	1200 m/z	Set Collision Cell RF	50.0 Vpp	Set Divert Valve	Waste

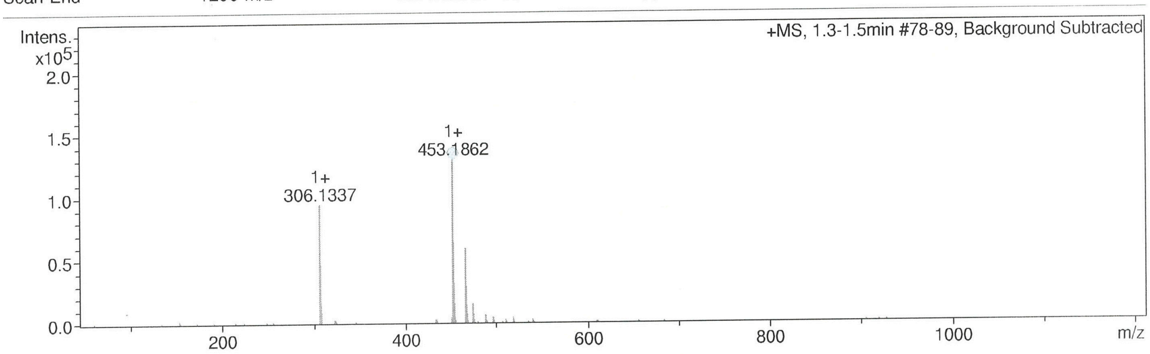

Meas. m/z	#	Ion Formula	m/z	err [mDa]	err [ppm]	mSigma
453.186198	1	C22H25N6O5	453.188094	1.9	4.2	8.8

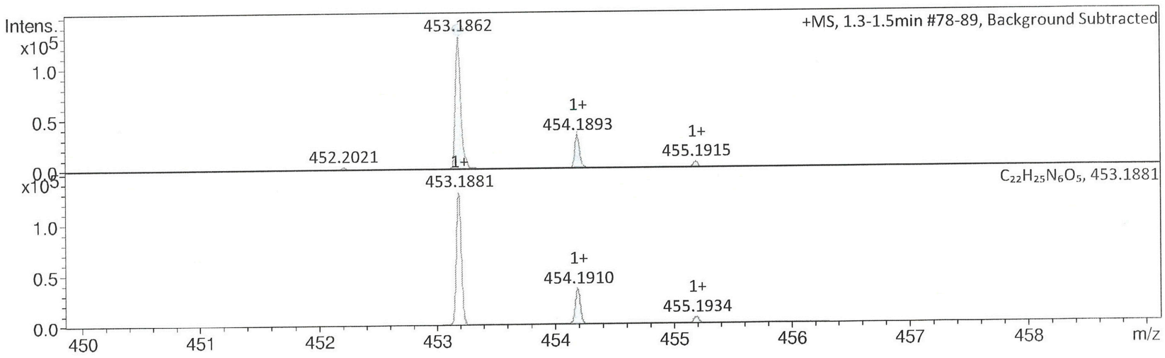

HRMS – 6

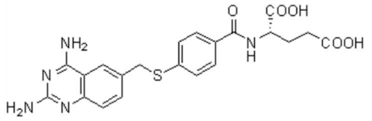

Acquisition Parameter

Source Type	ESI	Ion Polarity	Positive	Set Nebulizer	2.8 Bar
Focus	Not active	Set Capillary	4500 V	Set Dry Heater	180 °C
Scan Begin	50 m/z	Set End Plate Offset	-500 V	Set Dry Gas	9.0 l/min
Scan End	1200 m/z	Set Collision Cell RF	50.0 Vpp	Set Divert Valve	Waste

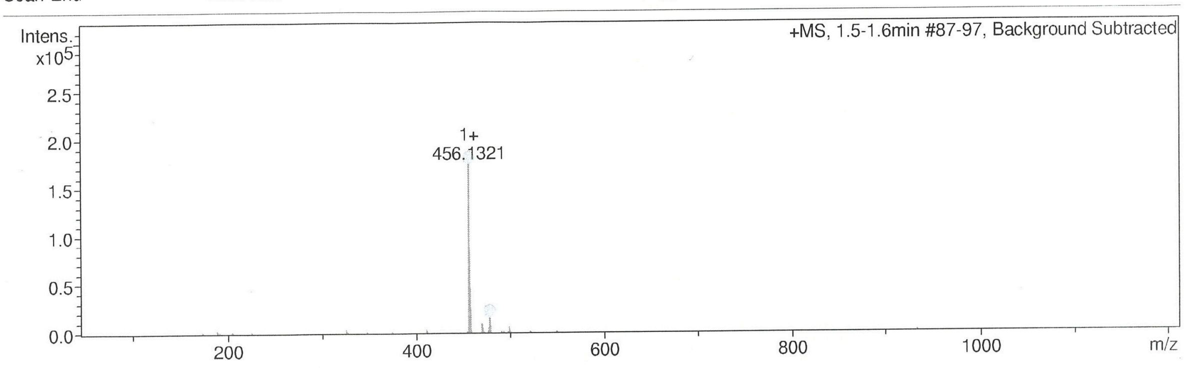

Meas. m/z	#	Ion Formula	m/z	err [mDa]	err [ppm]	mSigma
456.132087	1	C21H22N5O5S	456.133616	-1.5	-3.4	8.4
478.113320	1	C21H21N5NaO5S	478.115560	-2.2	-4.7	12.3

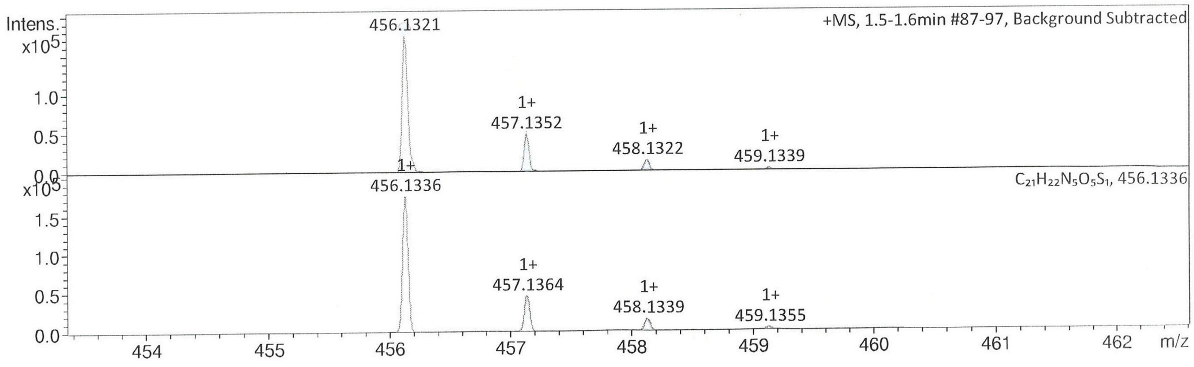

HRMS – 7

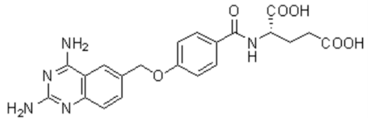

Acquisition Parameter

Source Type	ESI	Ion Polarity	Positive	Set Nebulizer	2.8 Bar
Focus	Not active	Set Capillary	4500 V	Set Dry Heater	180 °C
Scan Begin	50 m/z	Set End Plate Offset	-500 V	Set Dry Gas	9.0 l/min
Scan End	1200 m/z	Set Collision Cell RF	50.0 Vpp	Set Divert Valve	Waste

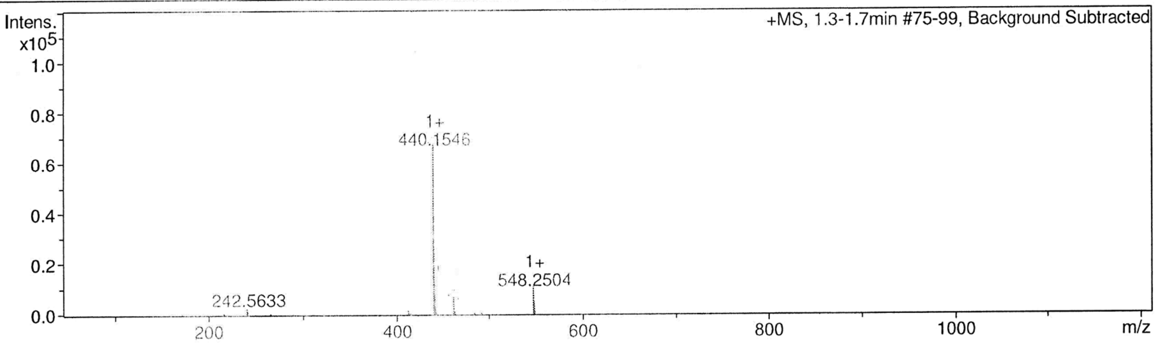

Meas. m/z	#	Ion Formula	m/z	err [mDa]	err [ppm]	mSigma
440.154647	1	C21H22N5O6	440.156460	1.8	4.1	11.0
462.136268	1	C21H21N5NaO6	462.138404	-2.1	-4.6	5.1

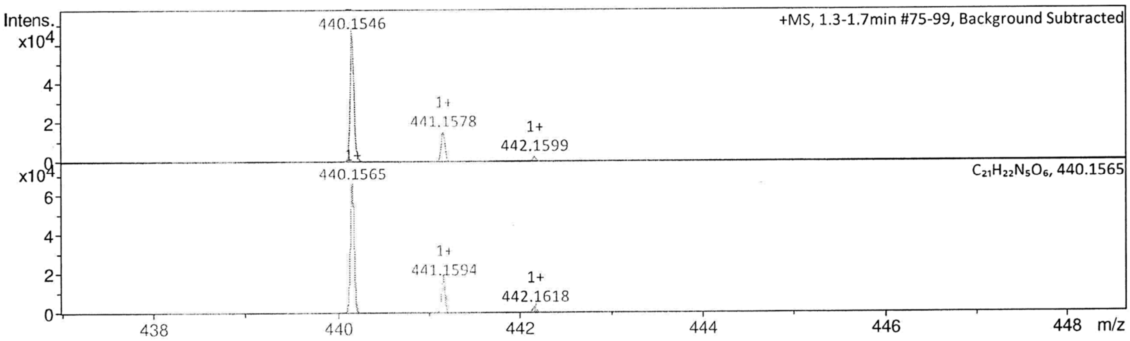

Chromatogram of methotrexate incubated for 12 h at 37 °C with human pooled S9 fractions. 7-Hydroxymethotrexate appears as expected.

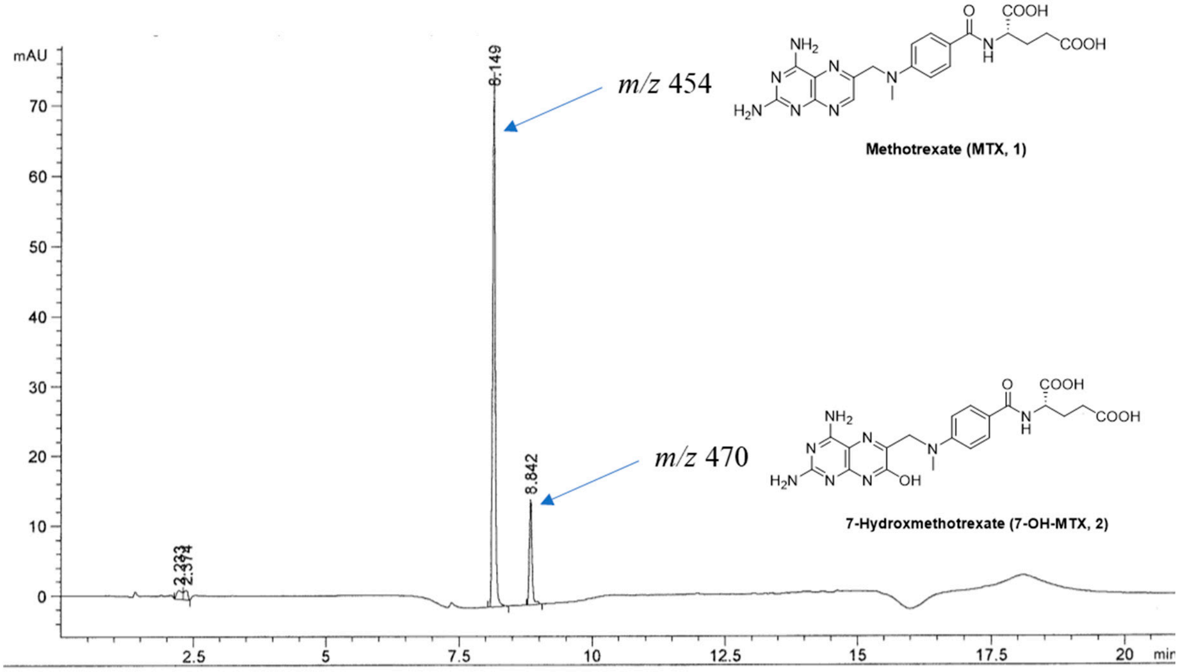

Chromatogram of 5,8-dideazamethotrexate (3) incubated for 12 h at 37 °C with human pooled S9 fractions.

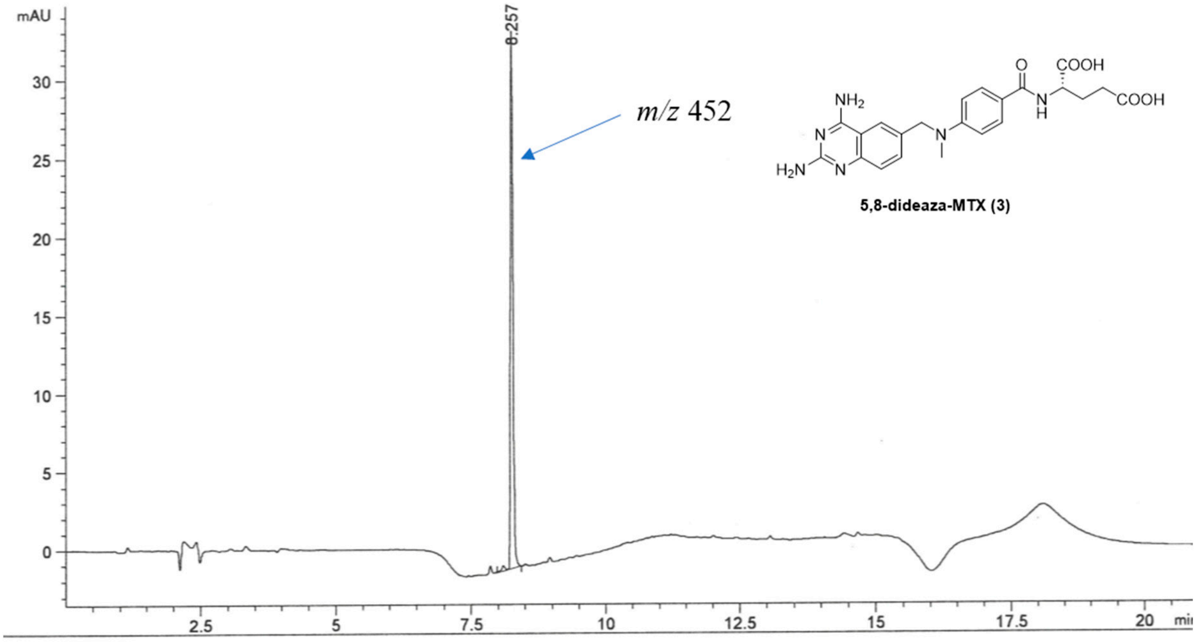

Chromatogram of compound 6 incubated for 12 h at 37 °C with human pooled S9 fractions.

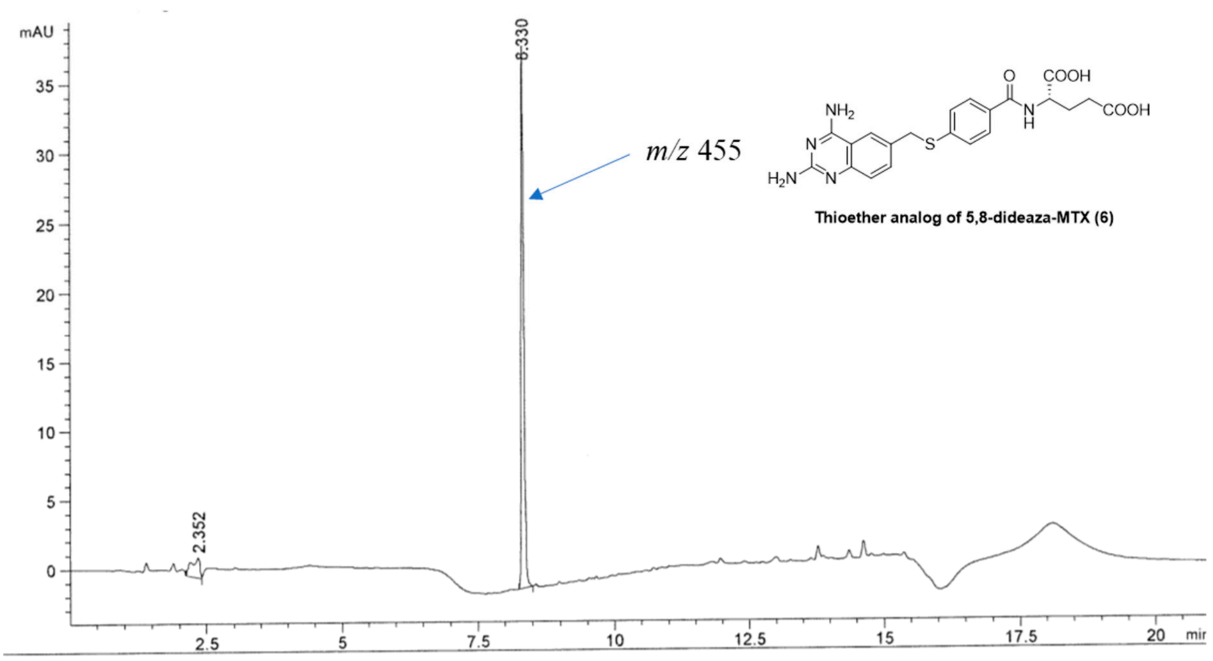

Chromatogram of compound 7 incubated for 12 h at 37 °C with human pooled S9 fractions.

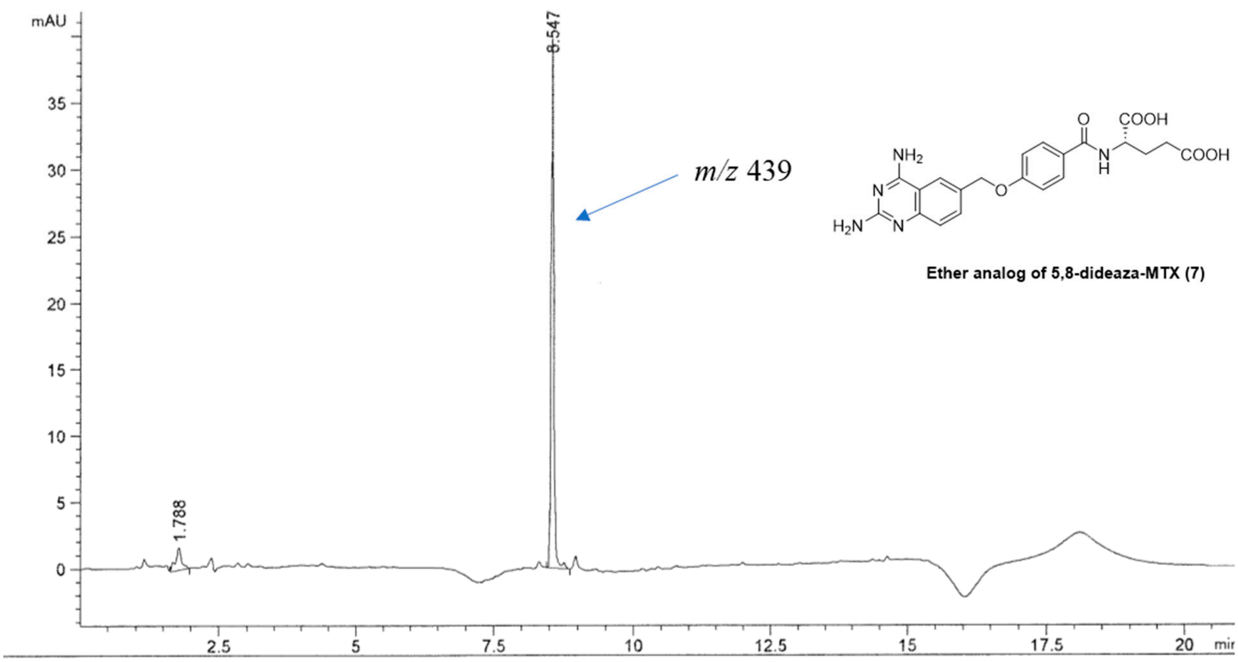

Supplement: Supplementary file 1 [file molecules-30-02772-s001.zip › molecules-3682184-supplementary.pdf]
